# Micro-homology intermediates: RecA’s transient sampling revealed at the single molecule level

**DOI:** 10.1093/nar/gkaa1258

**Published:** 2021-01-21

**Authors:** Andrew J Lee, Masayuki Endo, Jamie K Hobbs, A Giles Davies, Christoph Wälti

**Affiliations:** Bioelectronics, The Pollard Institute, School of Electronic and Electrical Engineering, University of Leeds, Woodhouse lane, Leeds LS2 9JT, UK; Institute for Integrated Cell-Material Sciences, Kyoto University, Yoshida-ushinomiyacho, Sakyo-ku, Kyoto 606-8501, Japan; Department of Physics and Astronomy, University of Sheffield, Houndsfield Road, Sheffield S3 7RH, UK; Bioelectronics, The Pollard Institute, School of Electronic and Electrical Engineering, University of Leeds, Woodhouse lane, Leeds LS2 9JT, UK; Bioelectronics, The Pollard Institute, School of Electronic and Electrical Engineering, University of Leeds, Woodhouse lane, Leeds LS2 9JT, UK

## Abstract

Recombinase A (RecA) is central to homologous recombination. However, despite significant advances, the mechanism with which RecA is able to orchestrate a search for homology remains elusive. DNA nanostructure-augmented high-speed AFM offers the spatial and temporal resolutions required to study the RecA recombination mechanism directly and at the single molecule level. We present the direct *in situ* observation of RecA-orchestrated alignment of homologous DNA strands to form a stable recombination product within a supporting DNA nanostructure. We show the existence of subtle and short-lived states in the interaction landscape, which suggests that RecA transiently samples micro-homology at the single RecA monomer-level throughout the search for sequence alignment. These transient interactions form the early steps in the search for sequence homology, prior to the formation of stable pairings at >8 nucleotide seeds. The removal of sequence micro-homology results in the loss of the associated transient sampling at that location.

## Graphical Abstract

**Graphical Abstract:**
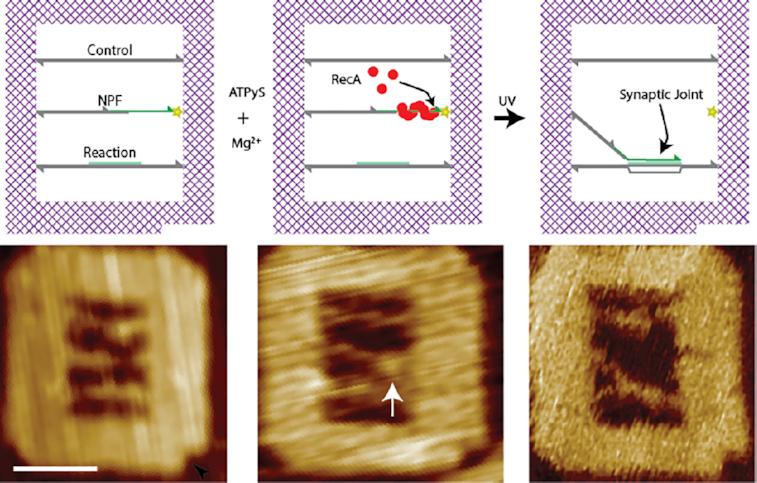
A schematic diagram depicting the homologous recombination reaction scheme within the DNA origami frame and associated HS-AFM micrographs. Left to right: the layout of the internal suspended DNA molecules (control, NPF and reaction) is shown in reference to an inbuilt polarity marker (black triangle). The RecA NPF (nucleoprotein filament) is formed on the 30 nt ssDNA region of the NPF DNA molecule and is connected into the DNA frame via a UV photocleavable linker (yellow star). Exposure to UV releases the NPF from its tether initiating homologous recombination. This culminates in the formation of a synaptic joint at the region of sequence homology (green) on the reaction DNA. HS-AFM image scale bar = 40 nm.

## INTRODUCTION

The process of homologous recombination—which exchanges one strand of a double-stranded DNA (dsDNA) with an identical or very similar sequence of single-stranded DNA (ssDNA)—is fundamental to all forms of life, maintaining genomic integrity, overcoming DNA lesions, rescuing DNA replication failures and affecting genetic diversity (1–3). Moreover, homologous recombination plays a significant role in the acquisition and horizontal transfer of antimicrobial resistance genes within bacterial populations.

Central to this pathway are the ubiquitous recombinases, such as the widely studied *Escherichia coli* Recombinase A (RecA), which catalyse the alignment and subsequent exchange of DNA strands at regions of sequence homology (4–6). While these proteins play a critically important role in a biological context, their programmable specificity and fidelity have made them appealing for use in bionanotechnological applications (7–10).

The RecA-mediated strand-exchange can be split up into three distinct steps. It begins with the polymerisation of RecA monomers onto single-stranded DNA in the presence of magnesium and ATP to form a right-handed helical nucleoprotein filament (NPF) (11–14). In this complex, each RecA monomer binds three nucleotides of the ssDNA through interactions with the backbone, leaving the nucleobases exposed at the core of the nucleoprotein complex (15).

The nucleoprotein filament subsequently probes dsDNA for regions of sequence homology between either of the constituent strands and the encapsulated ssDNA by forming a pre-synaptic joint (2,16). For this, the dsDNA substrate interacts with the NPF through the secondary binding site (binding site II) in the helical groove of the NPF (15,17,18). Here, the incoming dsDNA is transiently bound and a kink is induced in the backbone enabling the basepairs to be probed between the NPF-encapsulated ssDNA and the incoming dsDNA (17).

If base-pairing occurs—at a region of sequence homology—the originally transient NPF–dsDNA complex is stabilised and subsequent regions of the incoming duplex are probed in a processive manner, ultimately leading to the formation of a synaptic joint. It is noteworthy that the alignment of the dsDNA with the ssDNA encapsulated within the NPF, and the resulting strand-exchange, can occur without the input of chemical energy (2). In contrast, the final step of the recombination process—the disassembly of the NPF–dsDNA complex and thus the release of the new dsDNA duplex—requires hydrolysis of the RecA-bound ATP throughout the complex (19). Furthermore, while it has been shown that *in vitro* RecA can orchestrate the recombination of DNA molecules in isolation, the RecA-facilitated process is only one stage in the *in vivo* homologous recombination pathway which involves several other proteins to recruit and prepare the dsDNA as well as finalise the new pairing, and as such, RecA contains no known cleavage abilities (2).

The RecA-mediated homologous recombination process has been studied in its entirety, from formation of the nucleoprotein filament, alignment of homologous sequences, to strand exchange using an array of eloquent single molecule techniques, including magnetic tweezers (20–23), DNA curtains (24), tethered particle motion (25) and FRET-based systems (26–28). These studies reveal mechanistic subtleties that would otherwise be averaged out by more classical ensemble approaches. However, when paired with the plethora of biochemical (29,30) and structural studies (15) then a holistic understanding of the complex recombination process is formed. While these extensive studies provide invaluable contributions, they are mostly indirect observation of the homologous recombination process.

In contrast, our previous work focused on the investigation of the mode and specific interaction geometry of free RecA nucleoprotein filaments during a homology search, observed directly in real-time with high-speed atomic force microscopy (HS-AFM) (18). We showed that 1D facilitated diffusion along the dsDNA is utilised by the RecA NPF for the registration of DNA nucleotides at the ‘local’ level. Furthermore, this work has validated the interaction geometry derived through molecular dynamics modelling by Yang *et al.* (17) which proposed that the incoming dsDNA is being loaded into the binding site II in line with the right-handed helical groove of the nucleoprotein complex (18).

As such, direct observation with HS-AFM of the homologous recombination process arguably has a key role to play in providing novel insight where considerable gaps still remain in our understanding. Here, we directly observe the full RecA-orchestrated homologous recombination of DNA molecules within a DNA nanostructure and identify the transient (those which exist for <10 s) searching intermediates at the early stages of sequence alignment. We employ a DNA origami-based experimental platform (DNA frame) (18,31,32) to host the different DNA components required for homologous recombination, creating an environment that replicates a resected DNA break point as an initial substrate. The DNA frame not only provides the framework to host the reaction but also acts as a geometrical reference within which the orientation and relative positions of the interacting species can be measured, enabling the sequence-specific locations of short-lived interaction intermediates to be directly determined when observed in real-time with high-speed atomic force microscopy (HS-AFM).

## MATERIALS AND METHODS

### Materials

The DNA oligonucleotides used to form the internal DNA strands, including those which contained a photo-cleavable linker, were sourced from Integrated DNA Technologies (IDT) (Coralville, USA). The DNA oligonucleotide staples required to form the DNA frame were purchased from Eurofins Genomics (Tokyo, Japan). The M13mp18 ssDNA was purchased from Tilibit nanosystems GmbH (Munich, Germany). The sequences for all DNA used in this study are provided in supplementary information.

RecA protein (*E. coli*) was purchased from New England Biolabs (NEB; Ipswich, USA) at a concentration of 2 mg/ml and was diluted in a buffer containing 20 mM Tris-HCl (pH 7.5), 1 mM DTT, 0.1 mM EDTA and 5% glycerol to a 20 ug/ml working stock.

Adenosine 5-(γ-thio)triphosphate (ATPγS) tetralithium salt, made up to 5 mM; Mg(OAc)_2_, made up to 100 mM; Tris Acetate, made up to 100 mM, pH 7.4; and NiCl_2_, made up to 10 mM in deionised water, were all purchased from Sigma Aldrich (St. Louis, USA).

### Formation of DNA origami nanostructure

The DNA frame was designed using the caDNAno software (36,37). The DNA origami structure was formed from the collective self-assembly of 223 oligonucleotide staples (sequence list, Supplementary Table S1 & Supplementary Figure S10) and M13mp18 ssDNA scaffold. Structures were folded in 10 mM Tris acetate (pH 7.4), 10 mM Mg(OAc)_2_ and 1 mM EDTA by heating the mixture to 85°C followed by slowly cooling to 15°C at a gradient of –1°C min^−1^. The internal dsDNA strands were hybridised from their constituent oligonucleotides (sequence list, Supplementary Table S2) using the same heating/cooling protocol and were then incorporated into the DNA frame by adding a 5-fold excess to the DNA frame solution, heating the mixture to 45°C, followed by slowly cooling to 15°C at a gradient of –0.5°C min^−1^. Completed frames were purified using a Sephacryl S400 (GE Healthcare, Buckinghamshire, UK) size-exclusion matrix in a buffer containing 10 mM Tris-acetate, (pH 7.4), and 2 mM Mg(OAc)_2_, 1 mM EDTA to remove excess staples and unincorporated internal DNA.

### Polymerisation of RecA

RecA nucleoprotein filaments were formed within the DNA nanostructures as described previously (7). Briefly, RecA protein was introduced to the DNA origami at a ratio of 0.1–0.5 RecA monomers to three nucleotides (1 unit) of exposed ssDNA in the presence of 500 μM ATPγS, 10 mM Tris acetate (pH 7.4) and 2 mM Mg(OAc)_2_. The reaction was incubated at 37°C for 30 min.

### Recombination statistics

The reaction volume of DNA nanostructures containing RecA was adjusted to contain 10 mM Tris acetate (pH 7.4) and 10 mM Mg(OAc)_2_. The structures were exposed to UV (λ = 340 nm, 6.1 mW/cm^2^) for 10 s in order to break the photo-cleavable linker, thus releasing the nucleoprotein filament and enabling it to interact with the neighbouring internal dsDNA molecules. The reaction was allowed to proceed for 30 min at 37°C. The reaction volume was deposited upon Ni^2+^ pre-incubated mica and imaged with HS-AFM.

### In situ recombination

DNA nanostructures containing RecA were deposited upon Ni^2+^ pre-incubated mica, incubated for 20 mins, rinsed with deionised water and imaged using HS-AFM in buffer containing 10 mM Tris acetate (pH 7.4) and 10 mM Mg(OAc)_2_.

The nucleoprotein filament was released from the anchor following exposure to UV (λ = 340 nm, 6.1 mW/cm^2^) for 10 s *in situ*, and the subsequent nucleoprotein–DNA interactions followed at 22°C, while continually scanning with HS-AFM.

### HS-AFM imaging

All samples were imaged in tapping mode (amplitude modulation), in aqueous buffer, with a Bruker Dimension Fastscan AFM (Bruker Nanosurfaces, Santa Barbara, USA), using Fastscan D etched Si_3_N_4_ cantilevers (nominal spring constant = 0.25 N/m, Resonant frequency approximately 110 kHz in liquid) containing Si tips (nominal radius of curvature approximately 5 nm). Cantilevers were driven close to resonance under liquid and images were typically acquired at scan speeds between 38 and 60 Hz and a tapping amplitude of 10 nm. Typical images are acquired at 1 μm × 1 μm and 256 × 256 pixels giving a pixel size of ∼4 nm. Images were flattened by plane-fitting using the associated Nanoscope analysis software (Bruker Nanosurfaces, Santa Barbara, USA). ImageJ (http://rsbweb.nih.gov/ij/) was subsequently used to determine the position of the internal DNA strands throughout sequential images in a series by vectorising the AFM image as described in supplementary information (Figure S8).

## RESULTS AND DISCUSSIONS

### Formation of DNA frame and polymerisation of RecA

**Figure 1.**
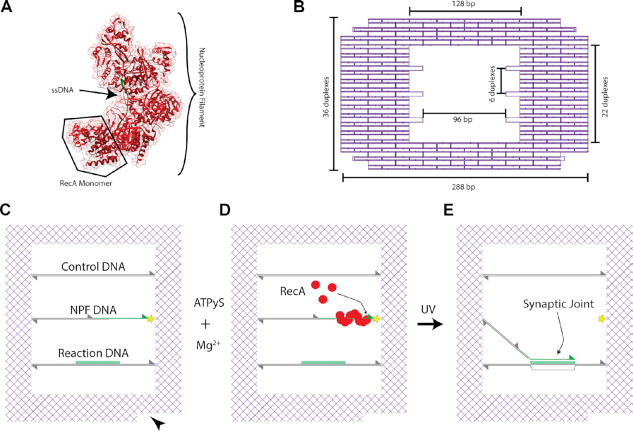
Overall experimental scheme. (**A**) A model structure of the RecA nucleoprotein complex generated from the crystal structure derived by Chen *et al.* (15) A partial turn of a nucleoprotein filament, containing five RecA monomers, is depicted with ssDNA (green) bound at its core. Generated from PDB entry 3CMT. (**B**) Detailed design of the DNA origami frame with the three sets of anchor points used to insert the internal suspended DNA molecules is shown schematically, with relevant dimensions indicated. (**C–E**) Schematic of the homologous recombination reaction scheme within the DNA origami frame. (**C**) The layout of the internal suspended DNA molecules (Control, NPF and Reaction DNA) is shown in reference to an inbuilt polarity marker (black triangle). The sequence directions are indicated by triangles at the 3′ termini. The NPF (nucleoprotein filament) DNA is partially formed from dsDNA with a 30 nt ssDNA region (green) at the 3′ termini which is connected into the DNA frame via a UV photocleavable linker (yellow star). The ssDNA which shares sequence homology with the green highlighted region of the Reaction DNA is the basis for the RecA NPF. A heterologous sequence is included as a Control DNA. (**D**) RecA is selectively bound to the ssDNA region forming an active nucleoprotein filament. (**E**) Upon UV exposure the NPF is released enabling the RecA-mediated homologous recombination reaction to proceed, culminating in a synaptic joint formed with the Reaction DNA. The DNA alignment in a triplex is indicated with RecA omitted for clarity.

The DNA frame contains an empty space at its centre (Figure 1B) within which three 128 basepair (bp)-long DNA molecules are incorporated parallel to each other to serve as the templates on which the single-molecule recombination experiments are conducted. The middle suspended DNA molecule (Figure 1C) is only partially double-stranded and contains a 30 nucleotide (nt) region of ssDNA at the 3′ termini upon which RecA can be selectively bound to form an active nucleoprotein filament (Figure 1D)—herein termed the NPF DNA. A 30 nt length was chosen due to the geometrical constrains of the DNA nanostructure system, which contains an ∼40 nm reaction window, and is consistent with the length scales used by other single molecule studies (24,33). RecA preferentially binds to ssDNA at low Mg^2+^ concentrations and hence its selectivity for this region of the structure can be modulated (2). The bottom dsDNA molecule features a 30 nt region on the upper strand which is homologous in sequence and parallel in orientation to the single-stranded region of the NPF DNA held within the DNA frame (Figure 1C)—herein this is called the Reaction DNA. In contrast, the sequence of the top dsDNA molecule does not feature the 30 nt homologous region and is utilised as an in-built heterologous control for recombination specificity (Figure 1C)—hence termed the Control DNA. Therefore, the active nucleoprotein filament formed on the termini of the NPF DNA is expected to create only a single specific recombination product upon completion of the reaction (Figure 1E). The orientation of the DNA frame, and therefore the identity of the different DNA molecules and the configuration of any recombination products, can be identified via the inbuilt polarity marker (Figure 1C, black triangle).

RecA has no known restriction activity (2) and hence a method for releasing the nucleoprotein filament from the DNA frame is required. Here, we use a UV photocleavable linker incorporated in the backbone of the NPF DNA (Figure 1C, yellow star) between the 3′ end of the ssDNA region and the anchor sequence used to hybridise it into the DNA frame. This enables cleavage and thus release of the nucleoprotein filament – initiating the recombination interaction—which can be triggered by exposure to UV light during the HS-AFM imaging.

**Figure 2.**
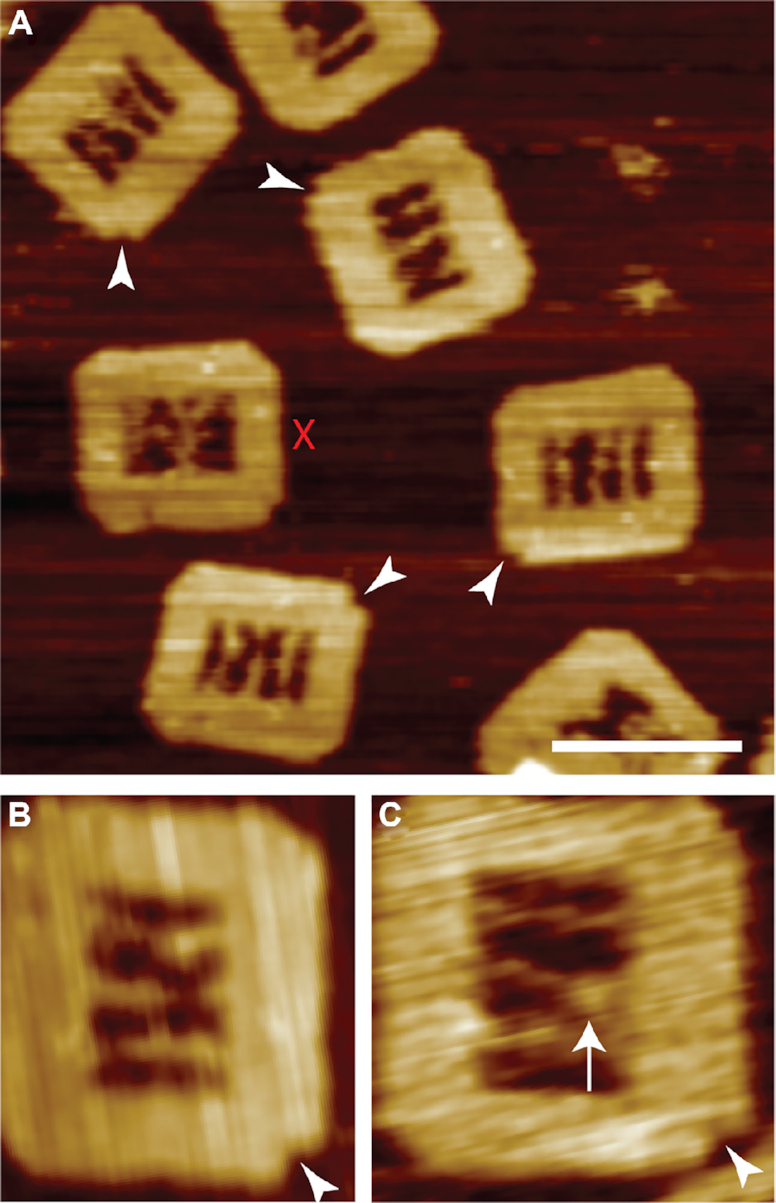
Assembly of the DNA frame and formation of RecA nucleoprotein filament on the NPF DNA. (**A**) AFM images of correctly assembled DNA frames including successful incorporation of all three internal DNA molecules. The polarity markers are indicated (white triangles). DNA frames with ambiguous arrangements of the internal DNA molecules are discounted (red cross) from subsequent analyses. Individual DNA origami frames are shown prior to (**B**) and following RecA nucleoprotein filament formation (**C**). The nucleoprotein filament is clearly visible on the ssDNA region (white arrow). Scale bar = 100 nm.

The successful assembly of the internal suspended DNA molecules and cleavage of the photocleavable linker was verified prior to (Supplementary Figure S1) and post incorporation (Figure 2) into the DNA frame by gel electrophoresis and AFM, respectively. From Figure 2A, B, clear identification of the polarity marker (white triangle) and inclusion of all three suspended DNA molecules within the central cavity can clearly be seen. The incorporation efficiency was found to be ∼60% when all the DNA molecules were introduced at a 5:1 (DNA:frame) ratio.

Despite the presence of a large single-stranded region at the end of the NPF DNA it was found to be able to bridge across the central void in the DNA frame and assemble to both anchor points, albeit with diminished efficiency when compared to the fully double-stranded Reaction and Control DNA.

We note that during HS-AFM imaging the DNA frame remains securely attached to the mica substrate, while the internal suspended DNA molecules remain clearly flexible—yet individually discernable—throughout sequential images. This suggests that they are not attached to the mica surface and therefore remain available for subsequent homologous recombination interactions. In particular, the region of ssDNA present within the NPF DNA is overtly flexible, in many cases observed as a gap in the DNA molecule due to the limited temporal response of the HS-AFM (Figure 2 B).

To study the homologous recombination reaction, a RecA nucleoprotein filament was formed on the ssDNA portion of the NPF DNA in the presence of the ATPγS. In order to ensure that the RecA only bound to the single-stranded portion and not to either the DNA frame or any of the suspended dsDNA molecules, the concentration of the protein was kept low at a ratio of RecA to three nucleotides of 0.1–0.5, and the Mg^2+^ was kept at 2 mM to maintain the single-strand specificity. Where these concentrations were exceeded unintended binding of RecA to other DNA components of the system were noted (Supplementary Figure S2).

From the AFM characterisation in Figure 2C, the presence of RecA at the expected location (white arrow) can clearly be seen. However, the structure differs slightly from the ones reported in earlier work (7,18,33,34), and in particular no significant difference in height between the dsDNA and the RecA nucleoprotein filament was observed. We speculate that at the concentrations of RecA required for this work, incomplete polymerisation occurs, and that although RecA is seen to bind to the DNA and is unambiguously demonstrated to be responsible for the homologous pairing later in this work (Supplementary Figure S4) it is likely to be a collection of small groups of bound monomers along the single-stranded region of the NPF DNA and that the extent of continuous polymerisation may be limited. This is also consistent with reports that 6–8 monomers are required to initiate polymerisation (2), and the 30 nt region employed here can only accommodate a maximum of 10 monomers.

### RecA-mediated recombination of DNA

To characterise the efficiency of the RecA-mediated recombination between the nucleoprotein filament on the suspended NPF DNA and the homologous region on the suspended Reaction DNA, the recombination reaction in the DNA frame was carried out in solution and the products characterised by AFM after deposition on the mica surface. Following the introduction of RecA, the formed nucleoprotein filaments were released with a 10 s UV exposure and allowed to interact for 30 min at 37°C in the absence of ATP hydrolysis. We note that the absence of the photo-cleavable linker prevents the release of the nucleoprotein filament during UV exposure (Supplementary Figure S3), confirming that nonspecific DNA damage is not responsible for the release and that the cleavage remains controllable. The 5′ end of the suspended NPF DNA remains tethered to the DNA frame through the dsDNA region of the molecule.

There is an equal probability for the tethered nucleoprotein filaments to interact with either the Control or Reaction DNA. However, we note that stable synaptic joints are only expected to form on the homologous region of the latter.

**Figure 3.**
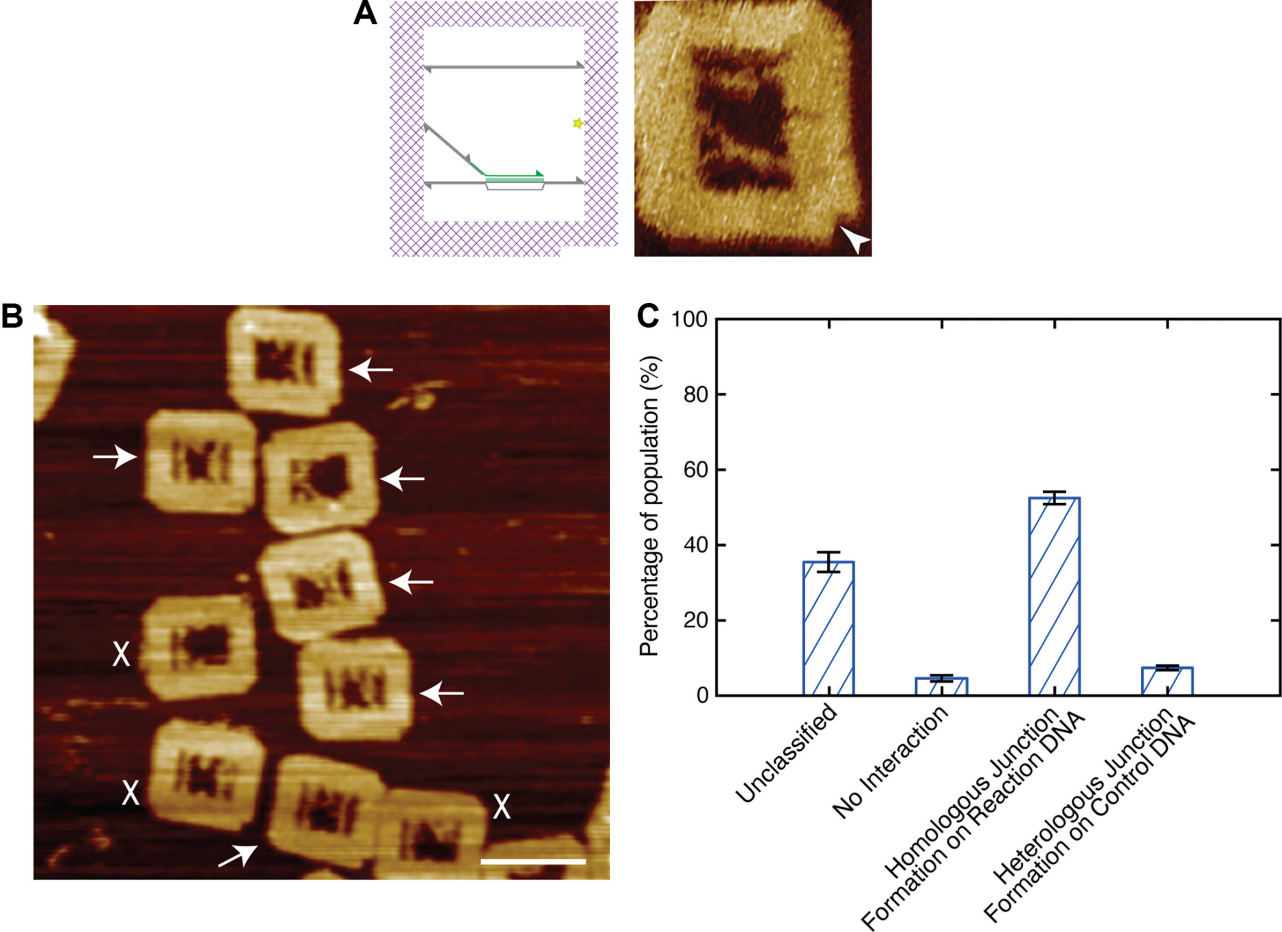
RecA-mediated homologous recombination. (**A**) A schematic diagram and atomic force micrograph of the DNA frame following successful homologous recombination. (**B**) A representative population of DNA frames following the recombination reaction. The successful recombined DNA frames are highlighted by white arrows. Frames where the internal DNA arrangements could not be identified clearly are discounted (white crosses). (**C**) Histogram showing the distribution of the recombined populations; number of frames analysed: 514. Scale bar = 100 nm.

Figure 3 shows the results of a typical recombination experiment. We observe successful RecA-mediated recombination, i.e. the formation of a synaptic joint on the Reaction DNA with an efficiency of 52.5 ± 1.6%. In contrast, the presence of stable joints formed with the heterogeneous Control DNA are found to exist with a much lower yield of only 7.4 ± 0.6%. A similar number, 4.6%, showed no RecA-mediated recombination. We note that around a third of all imaged DNA frames could not be analysed (35.5%) as they could not be classified. A number of representative examples of such unclassified frames is shown in Supplementary Figure S5 D. To rule out any geometrical bias in the difference of the yield, potentially imposed through the DNA frame, the positions of the Control and Reaction DNA within the frame were swapped. A similar ratio between homologous synaptic joints formed on the Reaction DNA, and incorrectly formed joints on the Control DNA, were found (Supplementary Figure S5).

### Characterising the observed synaptic joints

**Figure 4.**
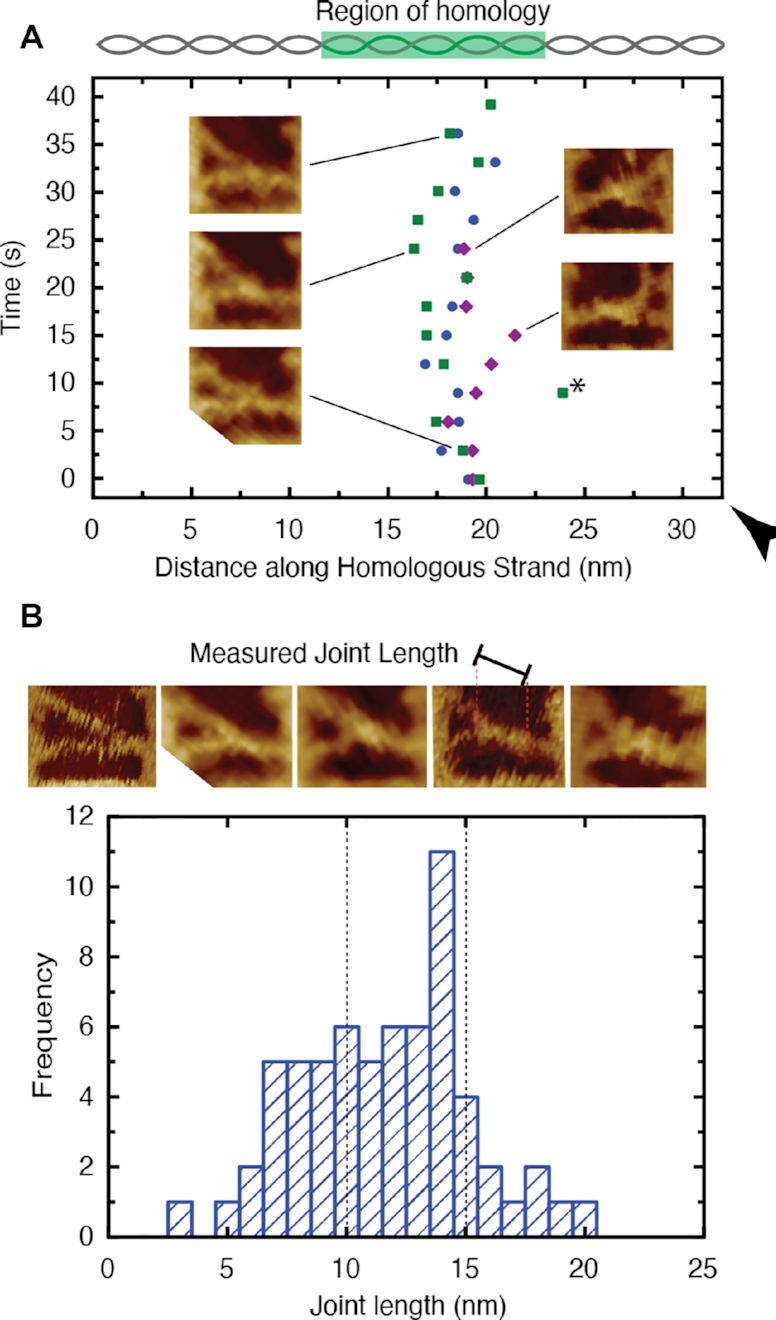
The stability and pairing length of the observed synaptic joints. (**A**) The location of three representative synaptic joints plotted as a function of position along the DNA molecule with reference to the polarity marker (black triangle). The joints are seen to be stable within the region of homology despite fluctuations of the DNA nanostructures. The insets show HS-AFM images of the joints at selected time-points. A large jump in position (black star) is noted as the result of a probe-induced image artefact (see Supplementary Figure S6). (**B**) Histogram depicting the distribution of measured nucleoprotein filament pairing lengths. The expected size range for the 30 nt nucleoprotein filament (10–15 nm) is indicated (dashed lines). AFM images of example synaptic joints of varying lengths and an indication of the measured length is provided above the histogram.

Despite the semi-flexible nature of the DNA frame support structure and the suspended DNA molecules, it remains possible to track the stable position of synaptic joints over time with HS-AFM. The position of the synaptic joints formed with the Reaction DNA can be measured via the distance along the DNA molecule. Figure 4A shows three representative joints which remain stably bound at the center of the designated region of homology (∼19 nm, ∼55 bp along the DNA strand from the end furthest from the polarity marker) (Figure 4A). This indicates that the observed synaptic joints are not transient intermediates but represent the correct formation of new base pairs, resulting in a strong complex able to resist the scanning probe of the HS-AFM. A notable outlier is highlighted (Figure 4A, black star) – this is the result of a probe-induced dislocation in the HS-AFM image, which occurs when the DNA frame moves slightly on the substrate during imaging (Supplementary Figure S6) thereby leading to an artefact.

Furthermore, the length of the paired complexes can be analysed to estimate the extent of sequence alignment within each synaptic joint. Figure 4 B shows a representative distribution of measured synaptic joint lengths. Given our limited knowledge of the extent to which RecA is polymerised upon the ssDNA section of the NPF DNA it is difficult to estimate the length of joint we would expect for full sequence pairing. However, it is known that RecA underwinds DNA by 1.5 times (2), and consequently it could be argued that a full 30 nt nucleoprotein filament would be expected to be 15.3 nm in length—the equivalent of 45 nt. However, in the present case, the RecA is unlikely to be fully polymerised and hence the underwinding is likely to be <1.5 times. Therefore, to estimate a lower limit of the nucleoprotein filament length, we assume the minimal case with no RecA underwinding present which yields a joint length of 10.2 nm. Here, we expect a distribution of RecA coverage on the ssDNA portion of the NPF strand and thus nucleoprotein filament lengths. Both limits are indicated in Figure 4 B and it can be seen that the observed distribution is indeed centered around this range with an average joint length of 11.1 ± 3.5 nm. It is notable that we observe a distribution of joint lengths below the lower bound suggesting that a proportion of nucleoprotein filaments are only partially aligned.

However, we note that given the observed variations of pairing length and bound RecA monomers, it is impossible to discern the exact sequence alignments from the length data alone in this work.

### In situ HS-AFM observation of homologous recombination

**Figure 5.**
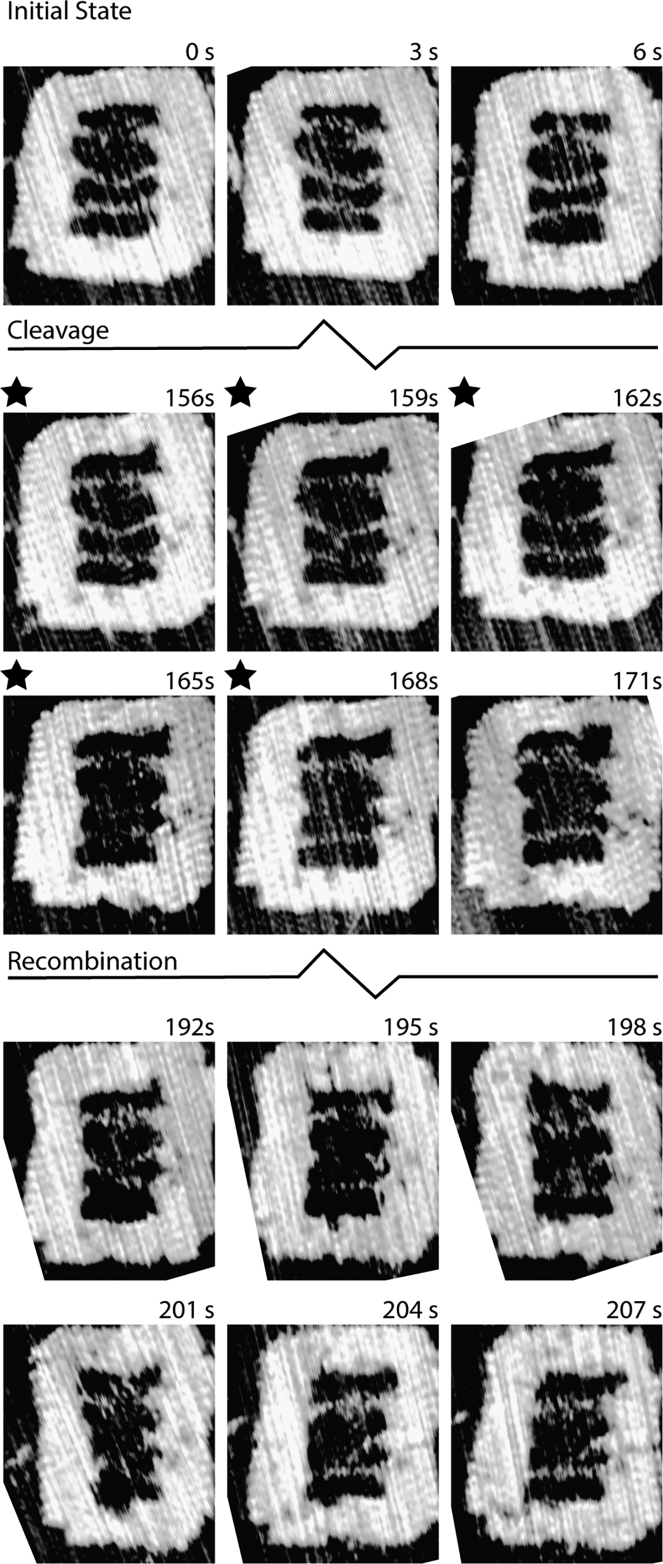
HS-AFM observation of RecA-mediated homologous recombination. The three suspended DNA molecules are intact within the DNA frame prior to UV exposure (black stars). Following photocleavage, the NPF DNA is released (165 s) and becomes difficult to track. A stable synaptic joint is formed at the region of homology (198 s) and persists through subsequent images indicating a stable complex. Individual images are extracted from Supplementary Movie S1.

The RecA-mediated recombination can be observed in real-time and at the single molecule level using HS-AFM (Figure 5 and Supplementary movie S1). At the beginning of the experiment, the NPF DNA containing the nucleoprotein filament at its end can be seen suspended across the central void of the frame clearly tethered on both ends. Immediately following cleavage of the UV photocleavable linker, the NPF DNA is released and becomes difficult to track due to its rapid movement relative to the speed of the scanning HS-AFM probe. The NPF has equal probabilities of reaching either the Reaction or the Control DNA and is therefore able to search all of the available sequence space. In all observed cases alignment is achieved at the region of sequence homology in the Reaction DNA resulting in a stable synaptic complex as intended. This is evident from the 198 s time-point in Figure 5 and persists at the same location throughout the remainder of the image series, revealing the first direct single-molecule observation of RecA-mediated homologous recombination.

Typically, the formation of a synaptic joint is observed within 30–40 s from initiation of the interaction upon release of the nucleoprotein filament from the DNA frame by UV cleavage (Figure 5, Supplementary Movie S1 & Supplementary Figure S7). Interestingly, in contrast the observation of a low number of heterologous pairings (those which occur with the Control DNA) when the reactions were conducted in solution prior to deposition on the mica surface (Figures 2-4), here no evidence for the formation of such heterologous pairings was seen when directly observing the homologous recombination via HS-AFM.

### The influence of micro-homology

Following RecA-mediated recombination events in real-time with HS-AFM reveals a landscape of transient sampling intermediates which appear at consistent locations between samples along the Reaction DNA in the absence of any artificial fixation agents. Such transient sampling events are defined as those interactions that exist for no more than 10 s in our experiments.

**Figure 6.**
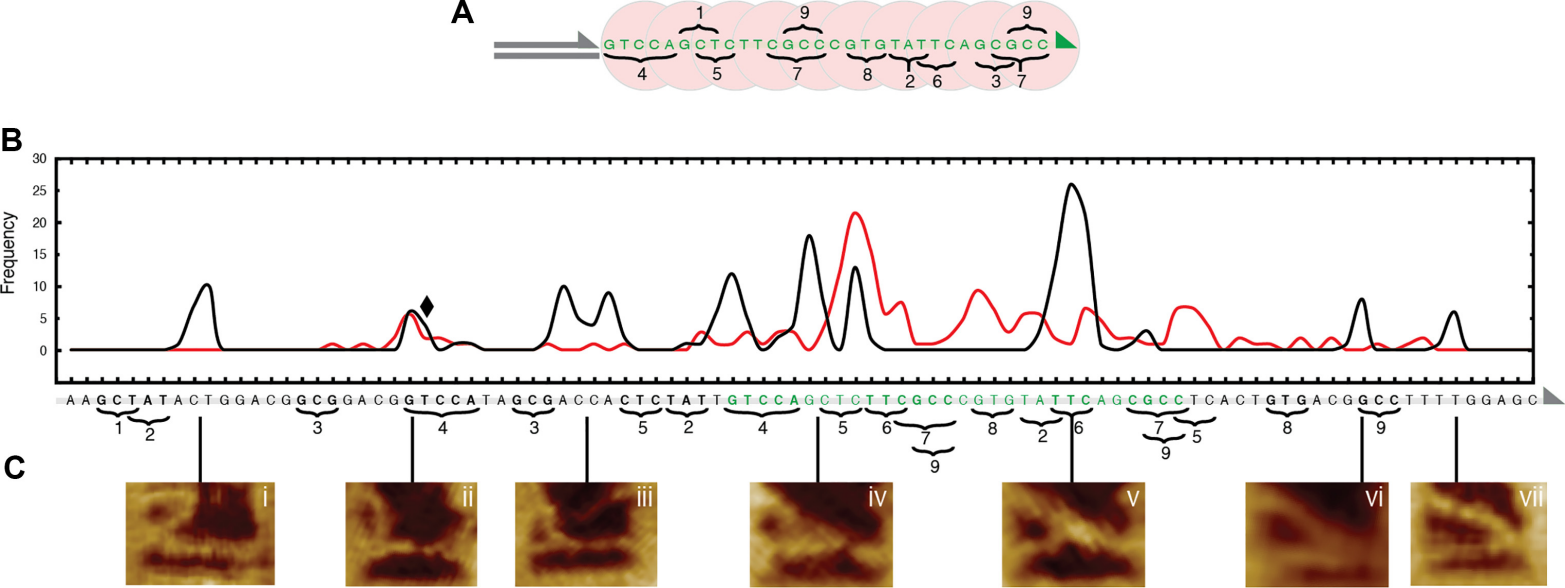
The influence of micro-homology upon the search for sequence homology. (**A**) The sequence of the RecA nucleoprotein filament is depicted and the instances of shared micro-homology 1–9 indicated. (**B**) Frequency distribution of measured transient sampling intermediates (black plot) with reference to the underlying DNA sequence. The designed region of homology is highlighted (green text). The existence of micro-homology shared between the dsDNA outside of the intended homologous sequence and the nucleoprotein filament are highlighted (1–9). When the micro-homology is purposefully disrupted the additional transient sampling intermediates disappear (red plot). When the micro-homology is switched from one sequence (4) to another (8) the corresponding transient sampling intermediate persists (black diamond). (**C**) AFM images of the corresponding characteristic transient sampling intermediates (i–vii) formed at various locations along the DNA sequence, albeit at a lower frequency than at the intended sequence. The orientation of the DNA sequences in A & B are indicated by triangles at the 3′ ends.

One RecA monomer of a NPF binds to three nucleotides of one strand of a double-stranded DNA with the same sequence as the 3 nt encapsulated in this RecA monomer. It is therefore reasonable to assume that for any observable interaction to occur between the NPF and the Reaction DNA, homologous sequences of at least 3 nt have to be present. When the underlying sequence of the Reaction DNA was analysed for instances of micro-homology shared between itself and the ssDNA region of the NPF DNA, nine domains—each 3–5 nt in length—were identified (Figure 6). In order to reconcile the existence of micro-homology with the observed transient sampling, the position of each interaction was determined by measuring the position on the Reaction DNA where the NPF DNA intersects with reference to the known dimensions of the DNA frame structure (Supplementary Figure S8). The corresponding position in the sequence of the Reaction DNA was determined by assuming a B-form DNA helix. All observed interactions are represented as a frequency distribution of measured interaction locations in Figure 6 B. To ensure stability of the nucleoprotein filaments, the duration of all experiments was kept significantly shorter than the half-life of ATPγS (∼120 mins). The measurement uncertainty is dominated by the flexibility of the DNA molecules within the DNA nanostructure, and has been estimated to be around 1–2 nm or 3–6 nt. We also note that the suspended NPF DNA is formed from three overlapping oligonucleotides and therefore retains a higher degree of flexibility than dsDNA (see Supplementary Figure S11), as it is able to bend and rotate around the nicks in the backbone. This limits a potential geometrical bias that the fixed tether may otherwise impose. It can clearly be seen that the NPF is not limited to a restricted distal region of, but in fact has access to, the entire suspended Reaction DNA. Interestingly, we find a strong correlation between the observed transient interactions (Figure 6C, i–vii) and the identified areas of micro-homology (Figure 6B, 1–9), indicating that transient interactions occur at areas of micro-homology as short as 3 nt.

Of the observed interactions, we find that the most common distribution occurs around the designed region of homology, where the potential for sequence alignment is the highest (Figure 6B, green text). In contrast, all other transient sampling observed outside of this region was found to be distributed with an even frequency suggesting that there is no particular sequence preference, but that the length of the sequence homology is important, consistent with previous observations (2). This was also the case where micro-homology was shared with the suspended Control DNA (Supplementary Figure S9).

In order to confirm further the specificity of the observed correlation, the micro-homology in domains 1, 2, 3, 5, 8 and 9 of the Reaction strand were all disrupted by shuffling of the nucleotide sequence. The resulting frequency distribution is shown in Figure 6B (red plot). From this it can be seen that the change in underlying sequence successfully leads to the loss of transient sampling intermediates being located in those regions (e.g. i, iii, vi and vii). In contrast, where the micro-homology domain is switched, in this case from domain 4 (GTCCA) to domain 8 (GTG), the transient sampling intermediate ii is still observed despite the sequence change (Figure 6B, black diamond).

This clearly demonstrates that the transient interactions during the RecA-mediated homology search observed with HS-AFM are governed by the sequence of the interacting dsDNA, and that sequences of micro-homology as short as 3 nt, corresponding to the interaction footprint of a single RecA monomer, lead to such transient interactions. These observed transient states therefore represent an early stage in the homology searching process, constituting an often ignored sampling step prior to the occurrence of stable pairing when a sequence of homology of 8 nt or longer has been located (24).

## CONCLUSIONS

Homologous recombination is critically important in maintaining genetic integrity across all forms of life. A breakdown of this recovery pathway results in deleterious cell activity such as carcinogenesis. Central to this process is the RecA family of recombinase enzymes which pair regions of sequence homology and enact a strand exchange in order to recover the damaged DNA. We have employed HS-AFM in conjunction with DNA origami nanostructures that display loosely-bound DNA molecules internally to visualise directly the dynamics of RecA-mediated homologous recombination *in situ* and at the single molecule level.

We have demonstrated that RecA-mediated homologous recombination can take place in the context of a DNA nanostructure with high specificity for the desired homologous sequence. In this setup, the initial spatial constraints on the active RecA nucleoprotein filament can be relaxed upon request, allowing the filament to interact with dsDNA within a determined radius. This opens up a range of avenues to exploit the capabilities of recombination enzymes *in vitro* for non-native applications such as bio-nanotechnology and molecular information processing.

Using our approach of HS-AFM augmented with DNA nano-reference structures, we demonstrate the direct observation and decode the transient interaction intermediates of the RecA-mediated homology searching process. Evidence of such initial transient contacts has been found previously through single molecule experiments (24–35), but their relevance has generally been overshadowed by the more stable pairings occurring at homologous sequences of 8 nt or more in length. Here we directly observe the transient sampling during the search for sequence homology, prior to stable synaptic joints being formed. Using the DNA nanostructure as a measurement reference we were able to identify the locations of the transient sampling interactions and correlate them with the occurrence of microhomology of three or more nucleotides in length. This demonstrates that RecA nucleoprotein filaments are able to transiently sample sequence space at the single RecA monomer level on short time scales (<10 s) in the early stages of a sequence alignment. Such contacts remain short-lived unless additional sequence alignment greater than 8 nt occurs which would lead to energetically stable homologous pairing, which would require ATP hydrolysis to resolve.

In conclusion, this work directly explores the early stages in the RecA-mediated homology searching mechanism by directly mapping interaction intermediates and thereby decoding the transient interaction space of RecA in real-time with HS-AFM. It provides the first direct observation of the initial transient basepair-driven homology sampling at the single-RecA-monomer-level, which is an early step in the homology searching mechanism.

## DATA AVAILABILITY

Data supporting this work can be accessed via the University of Leeds repository: https://doi.org/10.5518/945.

## Supplementary Material

gkaa1258_Supplemental_Files

## References

[1] West S.C. , Cassuto E., Howard-Flanders P. RecA protein promotes homologous-pairing and strand-exchange reactions between duplex DNA molecules. Proc. Natl. Acad. Sci. U.S.A. 1981; 78:2100–2104. 6941272 10.1073/pnas.78.4.2100 PMC319291

[2] Cox M.M. Regulation of bacterial RecA protein function. Crit. Rev. Biochem. Mol. Biol. 2007; 42:41–63. 17364684 10.1080/10409230701260258

[3] Seitz E. , Haseltine C., Kowalczykowski S. DNA recombination and repair in the Archaea.Advances inApplied Microbiology. Academic Press. 2001; 50:101–169. 10.1016/s0065-2164(01)50005-2 11677683

[4] Krejci L. , Altmannova V., Spirek M., Zhao X. Homologous recombination and its regulation. Nucleic Acids Res. 2012; 40:5795–5818. 22467216 10.1093/nar/gks270 PMC3401455

[5] Hong S. , Sung Y., Yu M., Lee M., Kleckner N., Kim K.P. The logic and mechanism of homologous recombination partner choice. Mol. Cell. 2013; 51:440–453. 23973374 10.1016/j.molcel.2013.08.008 PMC4049084

[6] Kawabata M. , Kawabata T., Nishibori M. Role of recA / RAD51 family proteins in mammals. Acta Med Okayama. 2005; 59:1–9. 15902993 10.18926/AMO/31987

[7] Sharma R. , Davies A.G., Wälti C. Directed assembly of 3-nm-long RecA nucleoprotein filaments on double-stranded DNA with nanometer resolution. ACS Nano. 2014; 8:3322–3330. 24593185 10.1021/nn405281s PMC4004295

[8] Braun E. , Keren K. From DNA to transistors. Adv. Phys. 2004; 53:441–496.

[9] Keren K. , Berman R.S., Buchstab E., Sivan U., Braun E. DNA-templated carbon nanotube field-effect transistor. Science. 2003; 302:1380–1382. 14631035 10.1126/science.1091022

[10] Keren K. , Krueger M., Gilad R., Ben-Yoseph G., Sivan U., Braun E. Sequence-specific molecular lithography on single DNA molecules. Science. 2002; 297:72–75. 12098693 10.1126/science.1071247

[11] Egelman E.H. , Stasiak A. Electron microscopy of RecA-DNA Complexes: two different states, their functional significance and relation to the solved crystal structure neutron scattering. Micron. 1993; 24:309–324.

[12] Egelman E.H. , Stasiak A. Structure of helical RecA-DNA complexes. Complexes formed in the presence of ATP-gamma-S or ATP. J. Mol. Biol. 1986; 191:677–697. 2949085 10.1016/0022-2836(86)90453-5

[13] Story R.M. , Weber I.T., Steitz T.A. The structure of the E. coli recA protein monomer and polymer. Nature. 1992; 355:318–325. 1731246 10.1038/355318a0

[14] Datta S. , Ganesh N., Chandra N.R., Muniyappa K., Vijayan M. Structural studies on MtRecA-nucleotide complexes: insights into DNA and nucleotide binding and the structural signature of NTP recognition. Proteins. 2003; 50:474–485. 12557189 10.1002/prot.10315

[15] Chen Z. , Yang H., Pavletich N.P. Mechanism of homologous recombination from the RecA-ssDNA/dsDNA structures. Nature. 2008; 453:489–484. 18497818 10.1038/nature06971

[16] Renkawitz J. , Lademann C.A., Jentsch S. Mechanisms and principles of homology search during recombination. Nat. Rev. Mol. Cell Biol. 2014; 15:369–383. 24824069 10.1038/nrm3805

[17] Yang D. , Boyer B., Prévost C., Danilowicz C., Prentiss M. Integrating multi-scale data on homologous recombination into a new recognition mechanism based on simulations of the RecA-ssDNA/dsDNA structure. Nucleic Acids Res. 2015; 43:10251–10263. 26384422 10.1093/nar/gkv883 PMC4666392

[18] Lee A.J. , Endo M., Hobbs J.K., Walti C. Direct single-molecule observation of mode and geometry of RecA-mediated homology search. ACS Nano. 2018; 12:272–278. 29202219 10.1021/acsnano.7b06208

[19] Reymer A. , Babik S., Takahashi M., Nordén B., Beke-Somfai T. ATP hydrolysis in the RecA–DNA filament promotes structural changes at the protein–DNA interface. Biochemistry. 2015; 54:4579–4582. 26196253 10.1021/acs.biochem.5b00614

[20] Forget A.L. , Kowalczykowski S.C. Single-molecule imaging of DNA pairing by RecA reveals a three-dimensional homology search. Nature. 2012; 482:423–427. 22318518 10.1038/nature10782 PMC3288143

[21] van der Heijden T. , Modesti M., Hage S., Kanaar R., Wyman C., Dekker C. Homologous recombination in real time: DNA strand exchange by RecA. Mol. Cell. 2008; 30:530–538. 18498754 10.1016/j.molcel.2008.03.010

[22] van Loenhout M.T.J. , van der Heijden T., Kanaar R., Wyman C., Dekker C. Dynamics of RecA filaments on single-stranded DNA. Nucleic Acids Res. 2009; 37:4089–4099. 19429893 10.1093/nar/gkp326 PMC2709578

[23] Peacock-Villada A. , Yang D., Danilowicz C., Feinstein E., Pollock N., McShan S., Coljee V., Prentiss M. Complementary strand relocation may play vital roles in RecA-based homology recognition. Nucleic Acids Res. 2012; 40:10441–10451. 22941658 10.1093/nar/gks769 PMC3488227

[24] Qi Z. , Redding S., Lee J.Y., Gibb B., Kwon Y., Niu H., Gaines W.A., Sung P., Greene E.C. DNA sequence alignment by microhomology sampling during homologous recombination. Cell. 2015; 160:856–869. 25684365 10.1016/j.cell.2015.01.029 PMC4344887

[25] Fan H.F. , Cox M.M., Li H.W. Developing single-molecule TPM experiments for direct observation of successful RecA-mediated strand exchange reaction. PLoS One. 2011; 6:e21359. 21765895 10.1371/journal.pone.0021359 PMC3134461

[26] Ragunathan K. , Joo C., Ha T. Real-time observation of strand exchange reaction with high spatiotemporal resolution. Structure. 2011; 19:1064–1073. 21827943 10.1016/j.str.2011.06.009 PMC3163668

[27] Ragunathan K. , Liu C., Ha T. RecA filament sliding on DNA facilitates homology search. Elife. 2012; 2012:e00067. 10.7554/eLife.00067 PMC3510455 23240082

[28] Cisse I. , Okumus B., Joo C., Ha T. Fueling protein-DNA interactions inside porous nanocontainers. Proc. Natl. Acad. Sci. U.S.A. 2007; 104:12646–12650. 17563361 10.1073/pnas.0610673104 PMC1937520

[29] Adzuma K. Stable synapsis of homologous DNA molecules mediated by the Escherichia coli RecA protein involves local exchange of DNA strands. Genes Dev. 1992; 6:1679–1694. 1516828 10.1101/gad.6.9.1679

[30] Adzuma K. No sliding during homology search by RecA protein. J. Biol. Chem. 1998; 273:31565–31573. 9813072 10.1074/jbc.273.47.31565

[31] Endo M. , Sugiyama H. Single-Molecule imaging of dynamic motions of biomolecules in DNA origami nanostructures using high-speed atomic force microscopy. Acc. Chem. Res. 2014; 47:1645–1653. 24601497 10.1021/ar400299m

[32] Yamamoto S. , De D., Hidaka K., Kyu Kim K., Endo M., Sugiyama H Single molecule visualization and characterization of sox2-pax6 complex formation on a regulatory DNA element using a DNA origami frame. Nano Lett. 2014; 14:2286–2292. 24660747 10.1021/nl4044949

[33] Lee A.J. , Sharma R., Hobbs J.K., Wälti C. Cooperative RecA clustering: the key to efficient homology searching. Nucleic Acids Res. 2017; 45:11743–11751. 28977583 10.1093/nar/gkx769 PMC5714135

[34] Lee A.J. , Szymonik M., Hobbs J.K., Wälti C. Tuning the translational freedom of DNA for high speed AFM. Nano Res. 2015; 8:1811–1821.

[35] Bell J.C. , Kowalczykowski S.C. RecA: Regulation and mechanism of a molecular search engine. Trends Biochem. Sci. 2016; 41:491–507. 27156117 10.1016/j.tibs.2016.04.002 PMC4892382

[36] Douglas S.M. , Dietz H., Liedl T., Högberg B., Graf F., Shih W.M. Self-assembly of DNA into nanoscale three-dimensional shapes. Nature. 2009; 459:414–418. 19458720 10.1038/nature08016 PMC2688462

[37] Douglas S.M. , Marblestone A.H., Teerapittayanon S., Vazquez A., Church G.M., Shih W.M. Rapid prototyping of 3D DNA-origami shapes with caDNAno. Nucleic Acids Res. 2009; 37:5001–5006. 19531737 10.1093/nar/gkp436 PMC2731887

