# Corrigendum to article “Micro-homology intermediates: RecA’s transient sampling revealed at the single molecule level’’

**DOI:** 10.1093/nar/gkab220

**Published:** 2021-03-22

**Authors:** Andrew J Lee, Masayuki Endo, Jamie K Hobbs, A Giles Davies, Christoph Wälti

**Affiliations:** Bioelectronics, The Pollard Institute, School of Electronic and Electrical Engineering, University of Leeds, Woodhouse lane, Leeds LS2 9JT, UK; Institute for Integrated Cell-Material Sciences, Kyoto University, Yoshida-ushinomiyacho, Sakyo-ku, Kyoto 606–8501, Japan; Department of Physics and Astronomy, University of Sheffield, Houndsfield Road, Sheffield S3 7RH, UK; Bioelectronics, The Pollard Institute, School of Electronic and Electrical Engineering, University of Leeds, Woodhouse lane, Leeds LS2 9JT, UK; Bioelectronics, The Pollard Institute, School of Electronic and Electrical Engineering, University of Leeds, Woodhouse lane, Leeds LS2 9JT, UK

The data DOI in this article (1) is incorrect. The authors wish to correct the Data Availability statement to:

## DATA AVAILABILITY

Data supporting this work can be accessed via the University of Leeds repository: https://doi.org/10.5518/945.

This change does not affect the results, discussion and conclusions presented in the article. The published article has been updated.
