# Supplementary material for: Micro-homology intermediates: RecA’s transient sampling revealed at the single molecule level
Source: Nucleic Acids Res. 2021 Jan 21;49(3):1426–35. doi: 10.1093/nar/gkaa1258 (PMC7897476; doi:10.1093/nar/gkaa1258)
Supplement: gkaa1258_Supplemental_Files [file gkaa1258_supplemental_files.zip › RecA_Recom_Supp_Info_R2.pdf]

Micro-homology intermediates: RecA's transient sampling revealed at the single molecule level

Supplementary Information

Andrew J. Lee [1], Masayuki Endo [2], Jamie K. Hobbs [3], Giles Davies [1] & Christoph Wälti [1]**

[1] Bioelectronics, The Pollard Institute, School of Electronic and Electrical Engineering, University of Leeds, Woodhouse lane, Leeds, LS2 9JT

[2] Institute for Integrated Cell-Material Sciences, Kyoto University, Yoshida-ushinomiyacho, Sakyo-ku, Kyoto 606-8501, Japan

[3] Department of Physics and Astronomy, University of Sheffield, Houndsfield Road, Sheffield, S3 7RH

*To whom correspondence should be addressed: A.lee@leeds.ac.uk. Correspondence may also be addressed to C.walti@leeds.ac.uk

Table of Contents

- Validation of suspended DNA molecule assembly and cleavage
- Excess RecA polymerisation
- UV exposure of non-photocleavable suspended NPF DNA
- Recombination in the absence of RecA
- Influence of position of Reaction vs Control DNA
- Probe-induced image dislocation artefacts
- Movie showing the synaptic joint formation
- Additional real-time recombination images
- Measurement of transient joint locations using DNA nanostructure
- Micro-homology mapping on the Control DNA
- DNA frame design
- DNA sequences
- References

Validation of suspended DNA molecule assembly and cleavage

The successful assembly of the suspended DNA molecules were validated using gel electrophoresis prior to incorporation into the DNA frames. From figure S1 it can be see that all three DNA molecules assemble successfully from their constituent oligonucleotides. The Control and Reaction DNA are formed from the hybridisation of two oligonucleotides at an offset such that ssDNA overhangs are formed that are subsequently utilised for incorporation into the DNA frame.

In contrast, the suspended NPF DNA (the nucleoprotein filament DNA), is formed from an arrangement of three overlapping oligonucleotides, with a large ssDNA region upon which RecA can be polymerised. The presence of the ssDNA regions results in multiple bands appearing within the PAGE gel. The correctly formed products are highlighted for all three suspended DNA molecules (figure S1, red box).

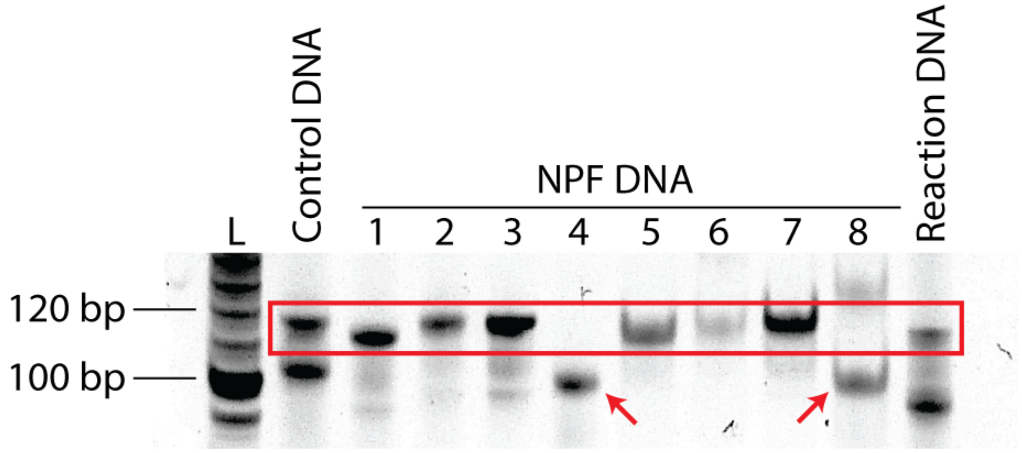

Figure S1. *Assembly and photocleavage with and without RecA of the internal suspended DNA molecules. A 10% Polyacrylamide gel demonstrating the formation of all three internal DNA (Control, NPF & Reaction) from their constituent oligonucleotide components. The correctly formed strands are indicated (Red box), with slight size variation evident due to the single-stranded terminal regions used to anchor the DNA into the DNA origami frame. NPF DNA lanes: 1 & 2) DNA without a photocleavable linker before & after exposure to UV, respectively. No truncation of the DNA occurs. 3 & 4) as previous with the inclusion of a photocleavable linker 19 bp from the 3' termini of the DNA. Truncation of the DNA occurs successfully (red arrow). 5 & 6) as 1 & 2 with the addition of RecA polymerised on the DNA. No truncation products evident following UV exposure (6). 7 & 8) as 3 & 4 with the addition of RecA polymerised on the DNA. Successfully truncation of the DNA following UV exposure occurs irrespective of the presence of RecA (8, red arrow). L = 10 bp ladder.*

In order to initiate the homologous recombination reaction *in situ*, a photocleavable linker was incorporated at 3' end of the NPF DNA between the region were RecA would be polymerised and the anchor sequence used to hybridise into the DNA frame. Upon exposure to UV this 3' terminal region containing the anchor sequence can be truncated releasing one end of the NPF DNA from the DNA frame. The activity of this photocleavable linker was investigated in the absence of the DNA frame in order to confirm the viability of this release mechanism (figure S1, red arrows). Truncation is shown to occur successfully even in the presence of RecA, which is liable to polymerising over the region containing the photocleavable linker. It is important

to note that all RecA was removed by digestion with Proteinase K prior to the DNA being run within the gel.

Excess RecA polymerisation

Selective polymerisation upon the ssDNA region only of the NPF DNA within the DNA frame is not trivial. Careful optimisation and use of low RecA concentrations – as noted in the main text – was required. Typically we used a ratio of 0.1 – 0.5 RecA to 3 nt (1 unit) of ssDNA in the presence of 500 μM ATPγS, 10 mM Tris acetate (pH 7.4) and 2 mM $Mg(OAc)_2$. The reaction was incubated at 37˚C for 30 minutes. With these ratios we consistently achieved selective polymerisation on the ssDNA portion of the NPF DNA (see figure 2C, main text). Where these ratios were exceeded we typically observed the unintended binding of RecA to parts of the DNA nanostructure and other DNA strands (figure S2).

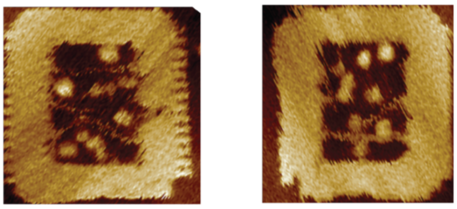

Figure S2. Unintended binding of excess RecA within DNA frame.

UV exposure of non-photocleavable suspended NPF DNA

Figure S3 depicts the DNA frame with all three suspended DNA molecules included during and after exposure to UV light using the same experimental procedures as in the rest of this study. Here, the photocleavable linker normally employed to release the NPF DNA from the frame at its 3' end is not included. No cleavage of any of the suspended DNA molecules, including the NPF DNA, was observed. This confirms that the release of the NPF DNA in the main text is due to the photocleaving of the linker and is hence controllable.

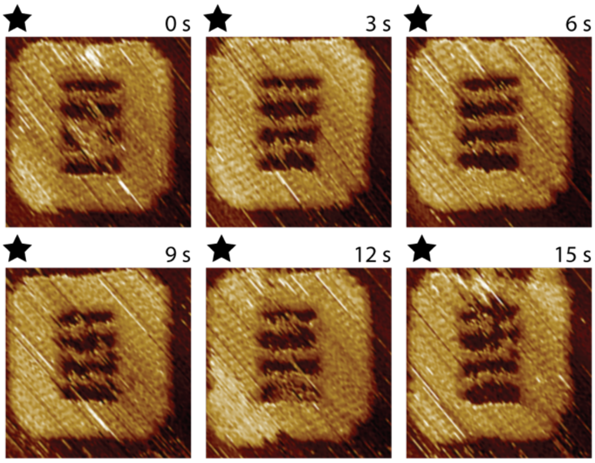

Figure S3. *UV exposure of the non-photocleavable linker central strand. UV exposure is indicated by the black stars.*

Recombination in the absence of RecA

Figure S4 shows a typical HS-AFM image sequence depicting a recombination experiment in the absence of RecA. The experiments were conducted in the same way as those described in the main text, but without the introduction of RecA. The DNA frames were subjected to the same incubation steps despite the absence of protein. From figure S4 it can be seen that under these conditions, upon UV cleavage, the central strand is seen to move rapidly within the frame tethered at the 5' terminal. No stable synaptic joints are observed, in contrast to what was observed in figure 5 & figure S5.

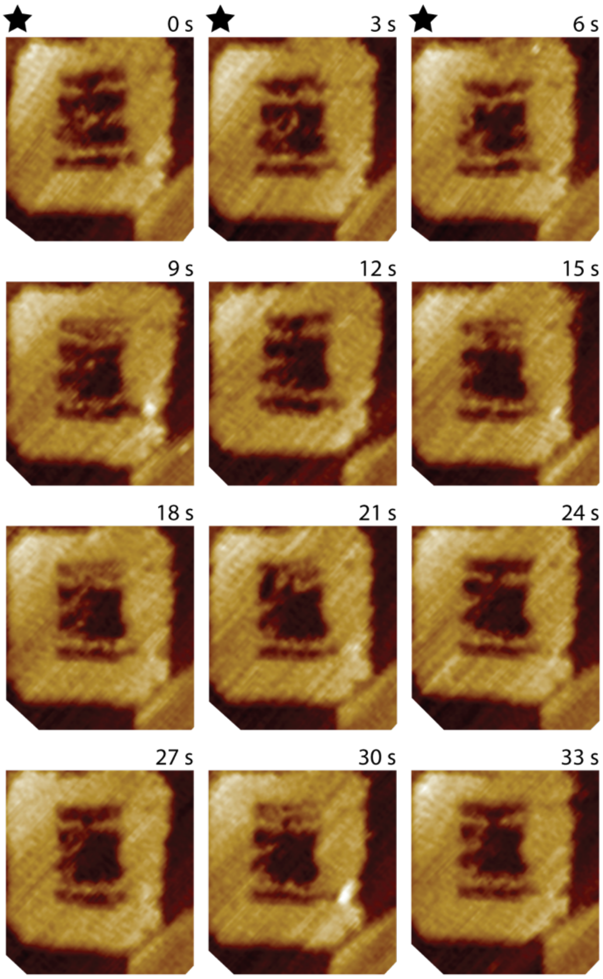

Figure S4. *Homologous recombination in the absence of RecA. Photocleavage of the suspended NPF DNA molecule leads to an increased flexibility of the NPF DNA. In contrast to the same experiment but in the presence of RecA, no synaptic joints are observed. UV exposure is indicated by the black stars.*

Influence of position of Reaction vs Control DNA

Geometrical influences were investigated by comparing the homologous pairing efficiencies of two variations of the frame. Type I, the standard orientation as in the main text, and type II where the suspended Reaction and Control DNA are swapped (figure S5). The ratio between synaptic joints formed between the Control and Reaction DNA is similar between type I and II frames, confirming the sequence specificity of the recombination. We note that there is a slight difference in the number of correct homologous synaptic joints between the frame variants (figure S5 C), with type I at 52.5 ± 1.6 % and type II at 36.4 ± 1.3 %, respectively. It is likely that this arises as a consequence of the variation in DNA frames which can be unambiguously classified from the images.

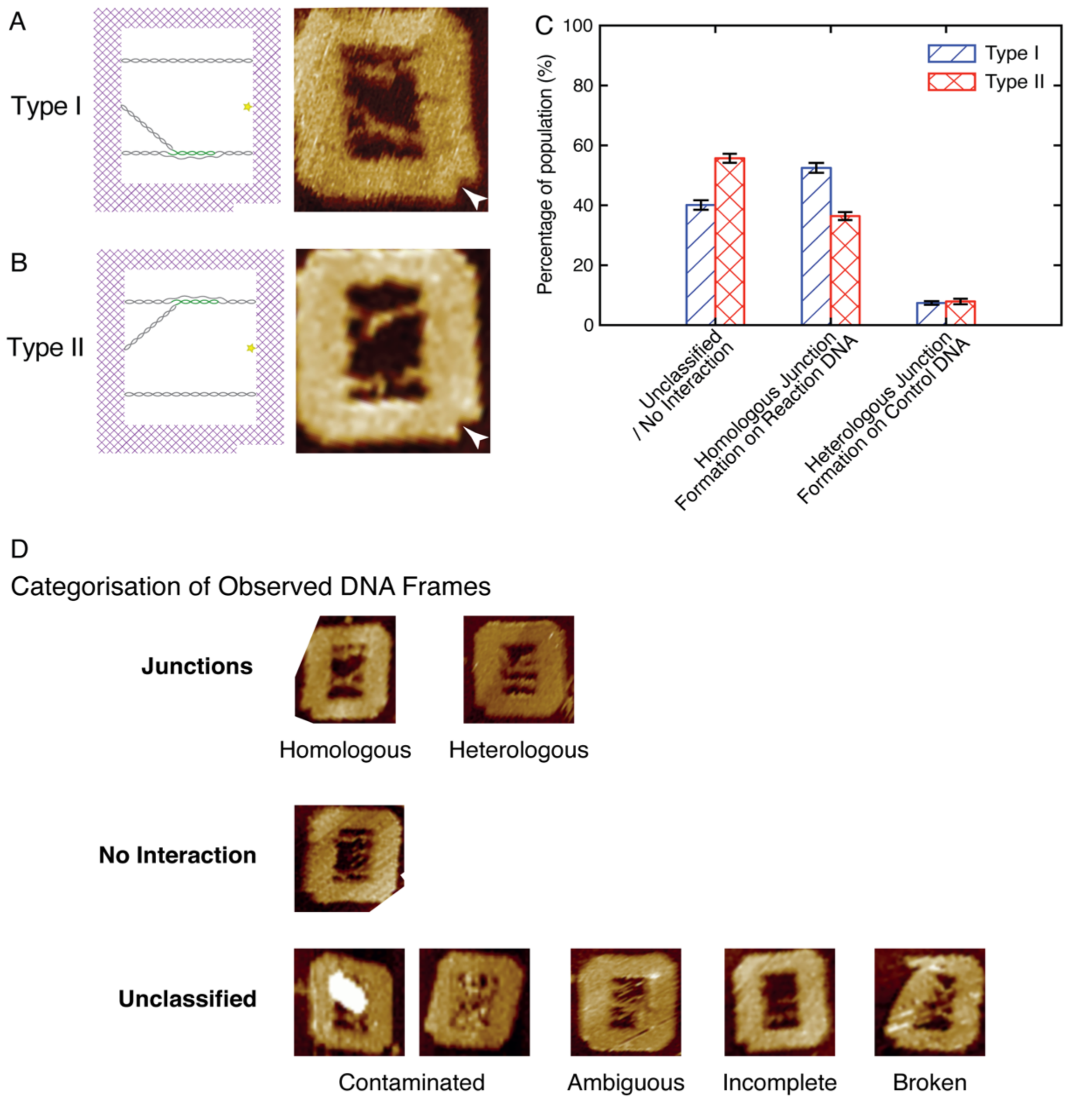

Figure S5. *RecA-mediated homologous recombination. Schematic and atomic force micrograph of the DNA frame following successful homologous recombination for type I (A) and type II (B) strand arrangements. C) Histogram showing the distribution of the recombined populations. D) Representative examples of variety of frames observed, including homologous and non-homologous (heterologous) junctions, frames where no interactions were observed, and unclassified frames.*

Probe-induced image dislocation artefacts

As with any microscopy technique, imaging artefacts can arise in HS-AFM. In HS-AFM, a rapidly scanning tip has to interact with the molecules to form an image, which can lead to artefacts. Figure S6 highlights an image artefact whereby the DNA frame has moved positions during the scan causing a dislocation to appear in the image leading to the disruption of the expected shape of the DNA nanostructure and the DNA strands within.

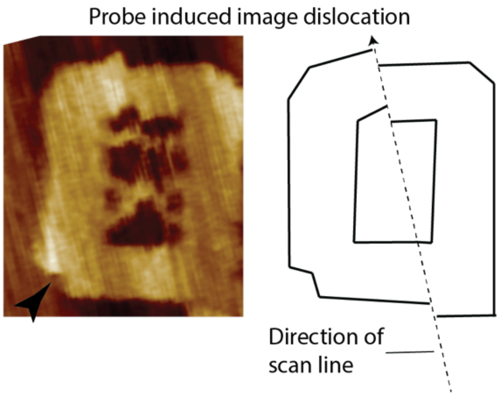

***Figure S6**. Dislocation imaging artefact due to movement of the DNA frame under the scanning HS-AFM probe.*

Movie showing the synaptic joint formation

Movie S1 shows a directly observed RecA-mediated recombination event within a DNA origami nanostructure. The suspended DNA molecules are tracked to ensure stability of the DNA structure. The recombination event is initiated by releasing the NPF DNA containing the RecA nucleoprotein filament via a 10 s UV exposure (white star). Immediately, the NPF DNA becomes very flexible and difficult to track until it begins to interact with the Reaction DNA eventually leading to the formation of a stable joint at 35 s. Scale bar = 20 nm.

Additional real-time recombination images

Figure S7 shows an additional observed recombination event analogous to figure 6 in the main text.

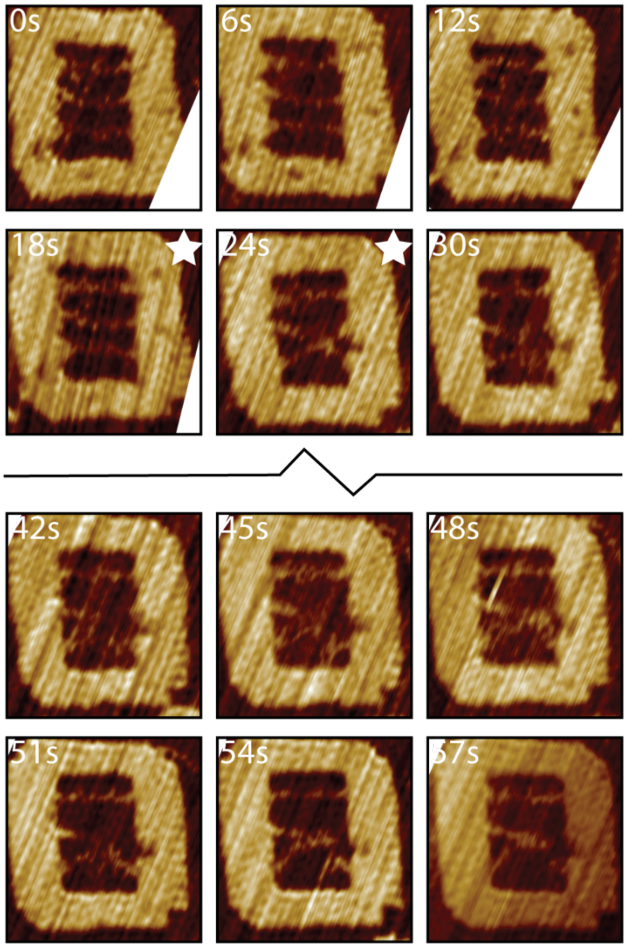

Figure S7. *HS-AFM observation of RecA-mediated homologous recombination. The three suspended DNA molecules are intact within the DNA frame prior to UV exposure (white stars). Following photocleavage, the NPF DNA is released (30 s) and becomes difficult to track. A stable synaptic joint is formed at the region of homology (48 s) and persists through subsequent images indicating a stable complex.*

Measurement of transient joint locations using DNA nanostructure

The position of all observe transient sampling intermediates were measured with reference to the designed dimensions of the DNA nanostructure as a molecular reference (figure S8). The raw image data was vectorised in line with our previous report[1] by fitting a worm-like-chain vector through the DNA molecules along the highest points of their cross section. This is conducted alongside fitting the DNA nanostructure cavity with a box with the designed dimensions of the structure. The measured distance is taken from the edge of the cavity box, farthest from the identified polarity marker, to the point of intersection between the two DNA vectors. The distance in nanometers is transformed into a sequence-position assuming a B-form DNA helix held in the designed position across the DNA nanostructure cavity window. Briefly, the distance in nanometers is divided by the number of nanometers per helical turn and multiplied by the number of base pairs in a single helical turn of the B-form helix. Taking into account the semi-flexible nature of the DNA strands and any breathing of the DNA nanostructure itself, this provides a reasonable and consistent method sufficient to successfully correlate the locations of observed interaction points and underlying DNA sequence.

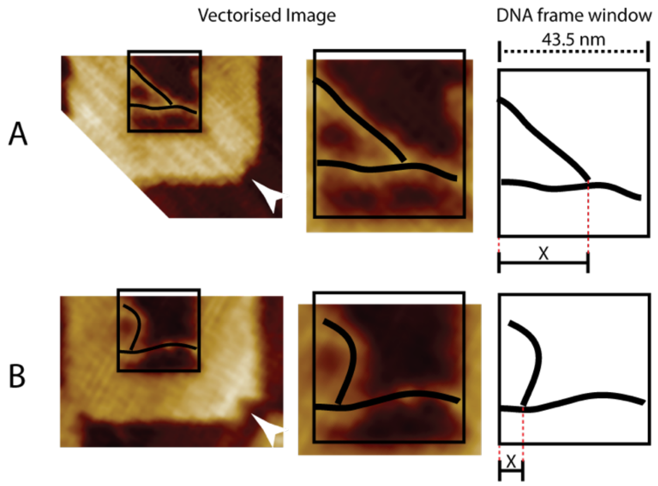

Figure S8. *Image vectorisation and interaction point measurement is depicted for two separate transient sampling intermediates (A & B). White arrow indicates DNA nanostructure polarity marker. The raw image is fitted with a vector representation of the internal cavity of the DNA nanostructure and the position of the DNA molecules within. The point of intersection between the incumbent DNA molecules is measured (X) with reference to the known dimensions of the internal cavity.*

Micro-homology mapping on the Control DNA

The transient sampling of micro-homology was mapped on the Control DNA for comparison with that for the Reaction DNA (figure S9). The mapping was conducted in the same way as described in the main text. From figure S9 it can be seen that there are 12 instances of micro-homology identified, largely grouped together into three separate regions which are associated with three distinctive transient joint positions. We note that the existence micro-homology on the Control DNA may contribute to the joints observed on the Control DNA in Figure 3.

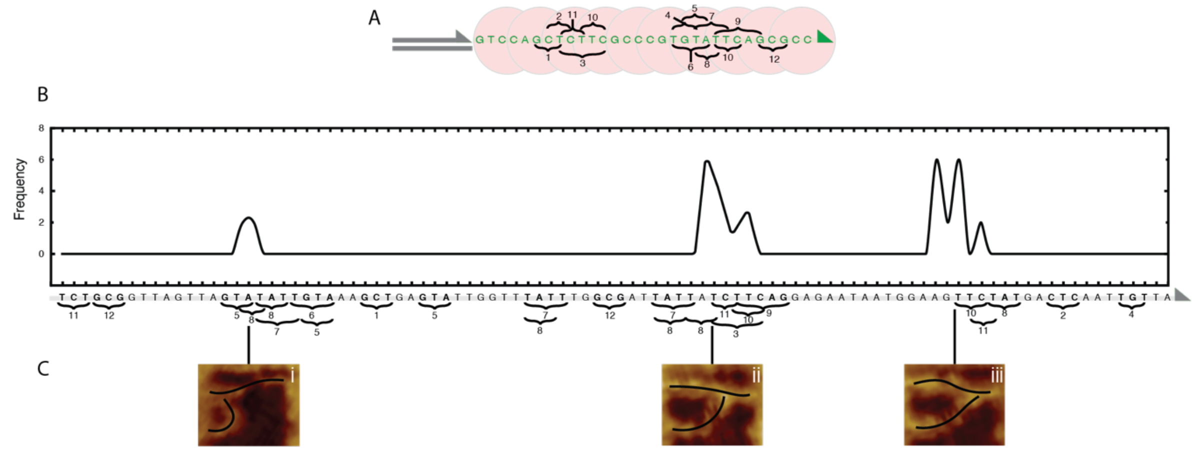

Figure S9. *The influence of micro-homology upon the search for sequence homology within the Control DNA. There are 12 tracts of micro-homology mapped between the RecA nucleoprotein filament (A) and the Control DNA (B). These are found to be grouped into three regions within the Control DNA. C) AFM images of the corresponding transient sampling intermediates (i – iii) formed along the Control DNA.*

DNA frame design

All the DNA oligonucleotide sequences utilised in this work are provided below alongside a schematic of the DNA frame design (figure S10). The sequences of the 223 staple oligonucleotides are provided in table S1. Each sequence is named with reference to its position in the DNA frame design (figure S10). The sequences for the constituent oligonucleotides of the suspended DNA molecules are provided in table S2. The ssDNA loops used to anchor the suspended DNA molecules are indicated by blue arrows and are separated by 94 bp across the internal cavity of the DNA nanostructure.

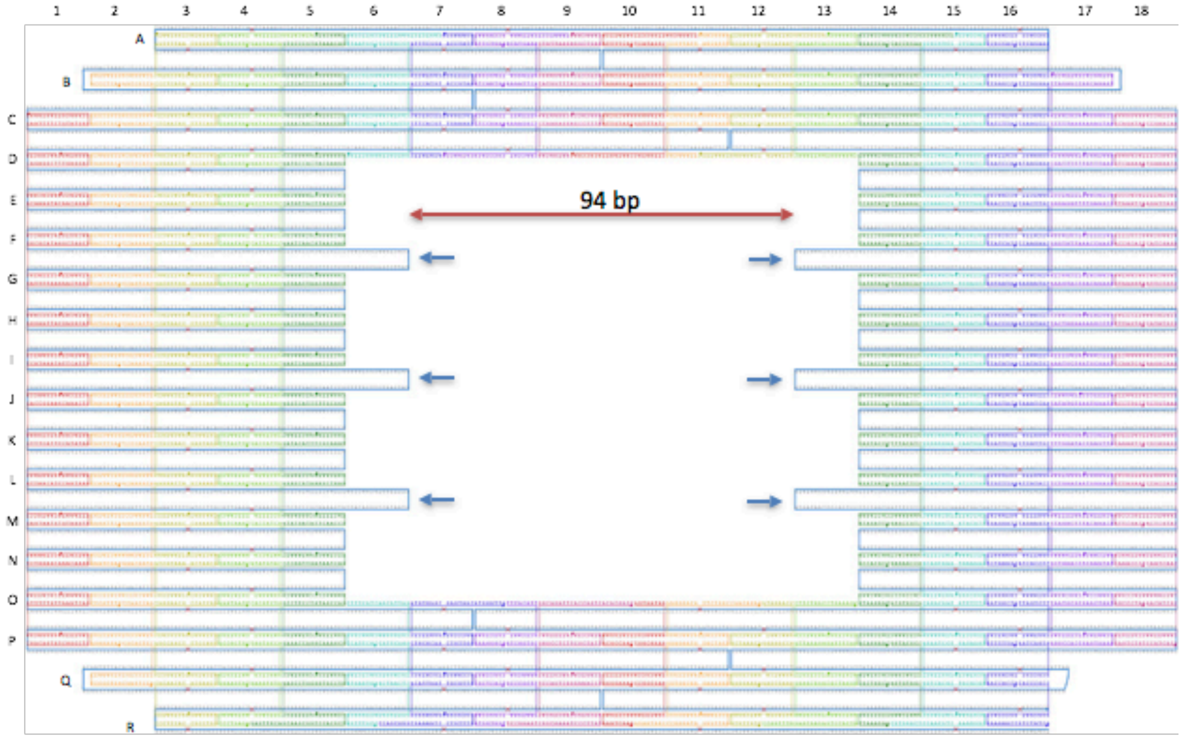

Figure S10. *Schematic diagram of M13mp18 ssDNA scaffold routing and position of the oligonucleotide staples that constitute the DNA frame. Staple oligonucleotides are referenced by grid positions, A – R and 1 – 18. The anchor positions for the internal strands are indicated (blue arrows).*

The internal arrangement of oligonucleotides that constitute the suspended DNA molecules is given in figure S11. The oligonucleotides are individually labeled with the respective sequences given in table S2.

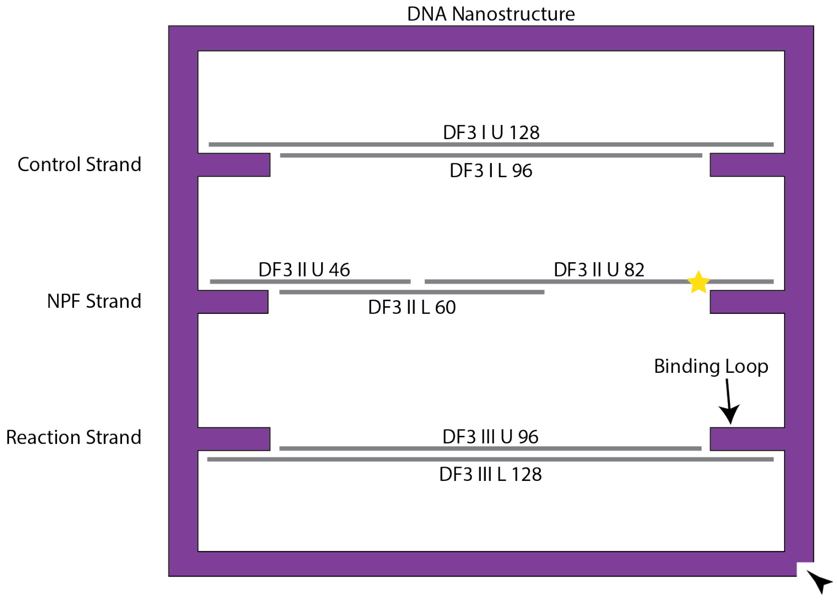

Figure S11. *Schematic representation of the arrangement of the constituent oligonucleotides that make up the suspended DNA molecules. The photo-cleavable linker is indicated by a yellow star, and the polarity marker of the DNA nanostructure by a black triangle.*

DNA sequences

Table S1. A list of the staple oligonucleotide sequences used to form the DNA frame from an M13mp18 ssDNA scaffold. The position of each oligonucleotide within the design is given with reference to figure S10.

Position Reference	Sequence (5'-3')
1C	ttttGAATTTTCTGTATGGGCCAGACGTTAGTAAATtttt
1D	ttttCAGAGGCTTTGAGGACAGGGTAGCAACGGCTAtttt
1E	ttttACAAAGTACAACGGAGTTATACCAAGCGCGAAtttt
1F	ttttGGCGCATAGGCTGGCTAACGGTGTACAGACCAtttt
1G	ttttGATTTTAAGAACTGGCGTGAATTACCTTATGCtttt
1H	ttttAGGAATTACGAGGCATGATACATAACGCCAAAtttt
1I	ttttTCATAAATATTCATTGCAATACTGCGGAATCGtttt
1J	ttttGACCGGAAGCAAACTCGCTTCAAAGCGAACCAtttt
1K	ttttGTTTCATTCCATATAAAGTACGGTGTCTGGAAtttt
1L	ttttATTAACATCCAATAAATACTAATAGTAGTAGCtttt
1M	ttttGAGTAATGTGTAGGTATTTAAATGCAATGCCTtttt
1N	ttttTCTGGAGCAAACAAGAGGTCATTGCCTGAGAGtttt
1O	ttttATTTTTGTTAAATCAGTTAAAATTCGCATTAAtttt
1P	ttttCGACAGTATCGGCCTCCAGTTTGAGGGGACGAtttt
2B	TCAGGAGGTTTAGTACACCCTCAG
2C	AACCGCCAAAGTTTTGTCGTCTTTATTTTGCT
2D	AAACAACTAGACAGCATCGGAACGTAAAGACT
2E	TTTTCATGCTTTGACCCCCAGCGAATTTGTAT
2F	CATCGCCTTGAAAGAGGACAGATGGACCTTCA

2G	TCAAGAGTTTCAACTTTAATCATTTCATTATA
2H	CCAGTCAGACATTCAACTAATGCAAGTAAGAG
2I	CAACACTATAGACTGGATAGCGTCAATCCCCC
2J	TCAAATGCTCGCGTTTTAATTCGACAACAGGT
2K	CAGGATTATTAAATATGCAACTAACAGTTGAT
2L	TCCCAATTAAGGTGGCATCAATTCTCATACAG
2M	GCAAGGCATAGAACCCTCATATATAAGATTCA
2N	AAAGGGTGTCTACAAAGGCTATCAGAATCGAT
2O	GAACGGTAAAACGTTAATATTTTGCTCATTTT
2P	TTAACCAAGTAACCGTGCATCTGCAGGAAGAT
2Q	CGCACTCCTTTCCTGTGTGAAATTTCCGCTCACAATTCCA
3A	CACCGTACAGTTTTAACGGGGTCGAGTGTACTGGTAATA
3B	ACGATCTACCCTCAGAACCGCCAAGGTGTAT
3C	GCAGCGAATTCAACAGTTTCAGCTTAGCGTA
3D	ACACTCATAGGAAGTTTCCATTACACCCTCA
3E	ACCAACTTGATAAATTGTGTCGAACACTAAA
3F	GGTTTAATAATCTTGACAAGAACCCGAACTG
3G	GGAATACCGACGTTGGGAAGAAACTTGAGAT
3H	AAAATGTTTCATAACCCTCGTTTGAGATTTA
3I	TTCAAATATTTAAACAGTTCAGAGTAATAGT
3J	AACATGTTGAGAGTACCTTTAATCGAAAGAC
3K	GAGCTGAACTGCGAACGAGTAGATGTAGCTC
3L	AAAATTTTAAGAATTAGCAAAATTGGGGCGC
3M	TTGAGAGAAGAAAGGCCGGAGACCAAGGATA
3N	TAAATTGTATCGTAAAACTAGCAAGCTATTT
3O	GGCGCATCTAGGAACGCCATCAACAAATATT
3P	CATAGCTGAGCCAGCTTTCCGGCTGTAGATG
3Q	GCTGATTGCACAACATACGAGCCATCATGGT
3R	CCCTTCACCGCCTGGGGGCAACA
4A	ATACATGGCTTTTGATGATACAGAGTGCCTT
4B	GAGTAACAAGTATAGCCCGGAATCCCTCAGA
4C	GCCACCACAGACAGCCCTCATAGGGAGTGAG
4D	AATAGAAACTTTTGCGGGATCGTAACGGGTA
4E	AAATACGTAAGAGGCAAAAGAATAATCCGCG
4F	ACCTGCTCTCAATCATAAGGGAACGGATATT
4G	CATTACCCGAGTAGTAAATTGGGAATCTACG
4H	TTAATAAAAAAGATTCATCAGTTACCAGACG
4I	ACGATAAAGTTTTGCCAGAGGGGAAACGAGA
4J	ATGACCATGATTAAGAGGAAGCCTGCTCCTT
4K	TTGATAAGTGCTGAATATAATGCTTTAGTTT
4L	GACCATTAAGCTATATTTTCATTTAAGCAAT
4M	AAAGCCTCGCCTTTATTTCAACGAGTCAAAT
4N	CACCATCAAATGCCGGAGAGGGTTGTCAATC
4O	ATATGTACGGAAGATTGTATAAGAAATAATT
4P	CGCGTCTGGATAGGTCACGTTGGACCGCTTC
4Q	TGGTGCCGGAGCTCGAATTCGTAGGAAGCAT

4R	AAAGTGTATTTCACCAGTGAGACCCCTGAGA
5A	TTGATATAGTGCCCGTATAAACAGTAAGCGTC
5B	CATTCCACCCTCATTTTCAGGGATCGAGAGGG
5C	AAAGGCCGGGAACAACTAAAGGAAGCCTGTAG
5D	TAAAACGAAATGCCACTACGAAGGAGGGAGTT
5E	GCAGACGGCATGTTACTTAGCCGGCACCAACC
5F	ACCAGAACAAATCAACGTAACAAAAACGAGGC
5G	ACAGGTAGACGAACTAACGGAACACGAGAAAC
5H	CAAAAGAAAACCAAAATAGCGAGAACATTATT
5I	ATCAAAAAAAATCAAAAATCAGGTGGCTTTTG
5J	AGCTTAATAGGTCATTTTTGCGGACGGATTGC
5K	ACCTGTTTGATACATTTCGCAAATTGGCTTAG
5L	CGGGAGAAAGAGCATAAAGCTAAAGGTCAATA
5M	GATAAATTATATGATATTCAACCGTACTTTTG
5N	CAAAAACACCCGGTTGATAATCAGTTCTAGCT
5O	CGTAATGGGCCTTCCTGTAGCCAGAAAAGCCC
5P	CGGGTACCGAAACCAGGCAAAGCGGGATTGAC
5Q	GTTTTTCTAAGCCTGGGGTGCCTAAGGATCCC
5R	GAGTTGCAGCAAGCGGTCCACGCTCCAGGGTG
6A	CAGTCTCTGAATTTACCGTTCCAGTTAATGCC
6B	CCCTGCCTGGCGGATAAGTGCCGTAGCAAGCC
6C	CAATAGGACAGTACAAACTACAACTTGCGAAT
6D	AATAATTTGGTCGCTGAGGCTTGC
6P	CTTTCATCAACATTAACGTGGGAACAAACGGCCCATTCGC
6Q	CATTCAGGTGCAGGTCGACTCTAGATGAGTGA
6R	GCTAACTCGTTTGCGTATTGGGCGGGTTTGCC
7A	CAGTACCAATTTCGGAACCTATTGGAAAGCG
7B	TTCGTCACACCCATGTACCGTAAGTTTTGCT
7C	ATATATTCTTTCACGTTGAAAATCACTGAGT
7O	GGATTCTCATGTGAGCGAGTAACATGGATTA
7P	TGCATGCCCTGCGCAACTGTTGGAACCCGTC
7Q	GAGAGGCGACATTAATTGCGTTGGCCAAGCT
7R	CCAGCAGGCGAAAATCCTGTTTGCGCGCGGG
8A	TAAATCCTCATTAAAGCCAGAATATTCTGAA
8B	ACATGAAAATTAGGATTAGCGGGATCAATAG
8C	AAAATTCAGTTTATTTTGTCACACTCCAAAA
8D	AAAAGGCTCAACCATCGCCCACGCATAACCG
8P	TTTACATTGCTCAATCGTCTGAAGAAGGGCG
8Q	ATCGGTGCAAAACGACGGCCAGTCGCTCACT
8R	GCCCGCTTTAATGAATCGGCCAAATGGTGGT
9A	AGAGAAGGGTATTAAGAGGCTGAGCAAACAAA
9B	CGGAATAATATGGTTTACCAGCGCACTCCTCA
9C	AATGACAACCAAAAGGAGCCTTTAAGACACCA
9O	ATTTTGACGGCAGATTCACCAGTCACACGACC
9P	GACGTTGTGGGCCTCTTCGCTATTATACCTAC
9Q	AGCTGCATTCCAGTCGGGAAACCTCCAGTCAC

9R	TCCGAAATCGGCAAAATCCCTTATGTCGTGCC
10A	TCAGACGATTGGCCTTGATATTCAATTTTCGG
10B	TCATAGCCGCGCGTTTTCATCGGCCAAAGACA
10C	AAAGGGCGTATAAAAGAAACGCAAATTGTATC
10D	GGTTTATCCTTGATACCGATAGTTGCGCCGAC
10P	AGTAATAAAAAAACGCTCATGGAAACGCCAGC
10Q	TGGCGAAATAACGCCAGGGTTTTCGGGGAAAG
10R	CCGGCGAACGATTTAGAGCTTGACAAATCAAA
11A	AGACTGTACCCTTATTAGCGTTTGAGGCAGG
11B	TGGCAACAACATTCAACCGATTGTTAGCGTC
11C	TTAAACAGAGCTTGCTTTCGAGGCATAAAGG
11O	GCAACAGGAAGGGACATTCTGGCCAACAGAG
11P	AAGTTGGGGGGGGATGTGCTGCACAGCCATT
11Q	GGAGCCCCCGTGGCGAGAAAGGAAGGCGATT
11R	AGAATAGCCCGAGATAGGGTTGACCCTAAAG
12A	GCCGCCAGCATTGACAGGAGGTTGCCATCTT
12B	TTCATAATGAATCAAGTTTGCCTAGGGAGGG
12C	AAGGTAAACGTAGAAAATACATAATAACCCA
12D	CAAGAATTGCTAATATCAGAGAGTGAATTTC
12P	ATAGAACCGAACAATATTACCGCGCTAAACA
12Q	GGAGGCCGAGAATCAGAGCGGGAAGGGAAGA
12R	AAGCGAAAAGCACTAAATCGGAAGTGTTGTT
13A	TAGCGACACAAAATCACCGGAACCCCAGAGCC
13B	TTAGCAAATATTGACGGAAATTATGTAATCAG
13C	CAGAGGGTAATTGAGCGAGTTAAGCCCAATAAGCAGTATG
13O	AATATCCACTTCTGACCTGAAAGC
13P	TCCTCGTTATTAAAGGGATTTTAGTTGCTGGT
13Q	TGCCGTAAGGAGCGGGCGCTAGGGACGTGCTT
13R	CCAGTTTGGAACAAGAGTCCACTAGGTCGAGG
14A	TCAGAGCCGCCACCAGAACCACCAAGAGCCAC
14B	CACCGGAACATCGATAGCAGCACCTCATTAAA
14C	GGTGAATTTAAGACTCCTTATTACTAAGAGCA
14D	AGAAACAAACACCCTGAACAAAGTGTTACAAA
14E	ATAAACAGAGAGCCTAATTTGCCACCGGTATT
14F	CTAAGAACGATATAGAAGGCTTATTAGAAACC
14G	AATCAATATAAGTCCTGAACAAGACATGTAAT
14H	TTAGGCAGTATTTAACAACGCCAAGTGTGATA
14I	AATAAGGCGTTTGAAATACCGACCACTACCTT
14J	TTTAACCTCGCTGAGAAGAGTCAAAATTTCAT
14K	TTGAATTAATTAATTACATTTAACCAGTACCT
14L	TTTACATCGATGAATATACAGTAAAGATGATG
14M	GCAATTCAAAAGAAACCACCAGAAATAGATTA
14N	GAGCCGTCGGAGCACTAACAACTAGCCACGCT
14O	GAGAGCCACACCGCCTGCAACAGTGTAAGAAT
14P	ACGTGGCAACTCAAACTATCGGCCACAGGAAC
14Q	GGTACGCCTTGACGAGCACGTATACGCTGGCA

14R	AGTGTAGCAAATCAAGTTTTTGGTTAAAGAA
15A	AATGAAACCCGCCTCCCTCAGAGCACCACCC
15B	GGCATGATATCACCGTCACCGACACGTCACC
15C	TTAACTGATGAAATAGCAATAGCAAAGAACT
15D	GTCTTTCCCCATATTATTTATCCCGGGAGAA
15E	GCAAATCAGCGAGGCGTTTTAGCTAACGAGC
15F	ACAATAGAATCGGCTGTCTTTCCAATAGCAA
15G	AATCGCCAAGGCATTTTCGAGCCGTTTATCA
15H	ATTTAATGGTTAAATAAGAATAATAATTGAG
15I	GATTAAGACCGGCTTAGGTTGGGTGACCTAA
15J	AAAACAAACCTTTTTTAATGGAAATAGCTTA
15K	TAACGTCAGGGAGAAACAATAACACATCAAG
15L	TGCGGAACTCAATATAATCCTGATTCAGGTT
15M	TATCTTTAAATAGATAATACATTTATCATTT
15N	AGTATTAAGCAGCAAATGAAAAAATCTAAAA
15O	GTAGAAGACAGACAATATTTTTGAGGCGGTC
15P	TGGTTGCTAGAATCCTGAGAAGTTTGCCTGA
15Q	CATCACCCGGTCACGCTGCGCGTGCGTACTA
15R	CGTGGACTCCAACGTCAAAGGGCACGTGAAC
16A	CGCCACCCTCAGAGCCCGCCACC
16B	CTCAGAACTTAGCAAGGCCGGAATTGAGCCA
16C	TTTGGGAAATAACGGAATACCCATATCTTAC
16D	CGAAGCCCGGGAAGCGCATTAGACAATCCAA
16E	ATAAGAAAGAATCTTACCAACGCGAACCTCC
16F	CGACTTGCATCATTACCGCGCCCTTATCATT
16G	CCAAGAACATGCAGAACGCGCCTAGTAATAA
16H	GAGAATATCTCAACAGTAGGGCTACACCGGA
16I	ATCATAATGTTAATTTCATCTTCTTATATAA
16J	CTATATGTCTTGAAAACATAGCGACAGTACA
16K	TAAATCAAAAGATGATGAAACAAGGATTCGC
16L	CTGATTGCGAAATTGCGTAGATTTTGTTTGG
16M	ATTATACTAAGTTTGAGTAACATTGAGGATT
16N	TAGAAGTAGAATTGAGGAAGGTTTCTAAAGC
16O	ATCACCTTGATAAAACAGAGGTGAATGGCTA
16P	TTAGTCTTTAGTAATAACATCACGTTTTTAT
16Q	AATCAGTGGCGCCGCTACAGGGCAACCACCA
16R	CACCCGCCGGGCGATGGCCCACTGAAAAACCGTCTATCA
17B	ACGCAATATTAGAGCCAGCAAAATAGTAGCACCATTACCA
17C	TAAAAACATTTTTAAGAAAAGTAACCGAGGAA
17D	TTTATCCTCGATTTTTTGTTTAACGAATAACA
17E	TCGTAGGAGGGAGGTTTTGAAGCCGCTACAAT
17F	TTCAGCTAGGGTATTAAACCAAGTTATTTTCA
17G	AGCCAACGAAAGTACCGACAAAAGACAACATG
17H	ATATTTTATACTAGAAAAAGCCTGCAGTATAA
17I	TAGAATCAAATGCTGATGCAAATTTTCAAAT
17J	AGCAAAAGTATATGTGAGTGAATAATTTTCCCT

17K	GAAATAAATTTGAATACCAAGTTAATTACCTG
17L	AATTTTAATCTGAATAATGGAAGGGTAAAACA
17M	GTTGAAAGTTAGACTTTACAAACAACGTTATT
17N	CAGCAGAAGCTGAACCTCAAATATAATCAACA
17O	TCTTTGATTAATGCGCGAACTGATCGAACCAC
17P	AGGCCACCGAGTAAAAGCAATACT
18C	ttttAGTTACCAGAAGGAAAGCAGATAGCCGAACAAtttt
18D	ttttGCAGCCTTTACAGAGAGTCAAAAATGAAAATAtttt
18E	ttttTGCTATTTTGCACCCATTAAATCAAGATTAGTtttt
18F	ttttACAAGCAAGCCGTTTTACCGCACTCATCGAGAtttt
18G	ttttCAGACGACGACAATAAGTAAAGTAATTCTGTCtttt
18H	ttttTTATACAAATTCTTACTTTAGTATCATATGCGtttt
18I	ttttGAACGCGAGAAAACTTCCAATCGCAAGACAAAtttt
18J	ttttTCGTCGCTATTAATTAACCTTGCTTCTGTAAAtttt
18K	ttttCGAATTATTCATTTCACAAAATCGCGCAGAGGtttt
18L	ttttTCAAAATTATTTGCACGTTAGAACCTACCATAtttt
18M	ttttTAAATCCTTTGCCCGAATTCGACAACTCGTATtttt
18N	ttttATCTGGTCAGTTGGCACAAACCCTCAATCAATtttt
18O	ttttCATTAAAAATACCGAAAGCCCTAAAACATCGCtttt
18P	ttttCAAATTAACCGTTGTAGAGTCTGTCCATCACGtttt

Table S2. *A list of oligonucleotide sequences used to form the suspended Control, NPF and Reaction DNA. All variants are denoted: Inverted = sequences used in Type II DNA frame, MiHo = Mico-homology removed. (*) The position of the photocleavable element (/**iSpPC**/) is depicted in line with the sequence.*

	Name	Sequence (5' - 3')
Control DNA	DF3 I L 96	TAACAATTGAGTCATAGAACTTCCATTATTCTCCTGAAGATAATAATCGCCAAATAAACCAATACTCAGCTTTACAATATACTAACTAACCGCAGA
Control DNA	DF3 I U 128	GCTGCTGATTCAGTCATCTGCGGTTAGTTAGTATATTGTAAAGCTGAGTATTGGTTTATTTGGCGATTATTATCTTCAGGAGAATAATGGAAGTTCTATGACTCAATTGTTACTAATTTACGAGCATG
Control DNA	DF3 I L 96 Inverted	AACATGTACTGGACGGAGGACGTGATCACGATACCACTTTATTGTCCAGCTCTTCGCCCGTGTATTCAGCGCCTCACTGAGACGGTTTCCTGGAGC
Control DNA	DF3 I U 128 Inverted	AAAATAATATCCCATCGCTCCAGGAAACCGTCTCAGTGAGGCGCTGAATACACGGGCGAAGAGCTGGACAATAAAGTGGTATCGTGATCACGTCCTCCGTCCAGTACATGTTATAAGGCTTGCCCTGA
Control DNA	DF3 I L 96 MiHo	TAACAATTGTGTCACAGTACTTCCATTATTCTCCTTAACATAACAATACCGAACTAAACCAAGTATCACGTTCTAAAATCTATAACTAACCACAGA
Control DNA	DF3 I U 128 MiHo	GCTGCTGATTCAGTCATCTGTGGTTAGTTATAGATTTTAGAACGTGATACTTGGTTTAGTTCGGTATTGTTATGTTAAGGAGAATAATGGAAGTACTGTGACACAATTGTTACTAATTTACGAGCATG
NPF DNA	DF3 II L 60	AGTATCTGTTCCACGAATCAGCGGTAAAGGTTGACTTAAATCGACCAGTAACAGGTGGCC
NPF DNA	DF3 II U 46	CTTTACCCTGACTATTGGCCACCTGTTACTGGTCGATTTAAGTCAA
NPF DNA	DF3 II U 82 Photo *	CCTTTACCGCTGATTCGTGGAACAGATACTGTCCAGCTCTTCGCCCGTGTATTCAGCGCCGTA**/iSpPC/**CGAATCATAGGTCTGAGAG
Reaction DNA	DF3 III L 128	GGAGCGGAATTATCATGCTCCAAAAGGCCGTCACAGTGAGGCGCTGAATACACGGGCGAAGAGCTGGACAATAGAGTGGTCGCTATGGACCGTCCGCCGTCCAGTATAGCTTCATTATGACCCTGTAA
Reaction DNA	DF3 III U 96	AAGCTATACTGGACGGCGGACGGTCCATAGCGACCACTCTATTGTCCAGCTCTTCGCCCGTGTATTCAGCGCCTCACTGTGACGGCCTTTTGGAGC
Reaction DNA	DF3 III L 128 Inverted	GGAGCGGAATTATCATATTGTTAACACAGTGTCATGAAGGTAATAAGAGGAATTGTATTGTTATGGCTTGATTTGGTTCATAGTGCAAGATTTTAGATATTGATTGGTGTCTCATTATGACCCTGTAA

DF3 III U 96 Inverted	AGACACCAATCAATATCTAAAATCTTGCACTATGAACCAAATCAAGCCATAACAATACAATTCCTCTTATTACCTTCATGACACTGTGTTAACAAT
DF3 III L 128 MiHo	GGAGCGGAATTATCATGCTCCAGGAAACCGTCTCAGTGAGGCGCTGAATACACGGGCGAAGAGCTGGACAATAAAGTGGTATCGTGATCACGTCCTCCGTCCAGTACATGTTCATTATGACCCTGTAA
DF3 III U 96 MiHo	AACATGTACTGGACGGAGGACGTGATCACGATACCACTTTATTGTCCAGCTCTTCGCCCGTGTATTCAGCGCCTCACTGAGACGGTTTCCTGGAGC

References

1 Lee AJ, Szymonik M, Hobbs JK, Wälti C. Tuning the translational freedom of DNA for high speed AFM. Nano Res 2015;8:1811--1821. doi:10.1007/s12274-014-0681-y.
